# Supplementary material for: Long-term physical and mental health outcomes of Ebola Virus Disease survivors in Kenema District, Sierra Leone: A cross-sectional survey
Source: PLOS Glob Public Health. 2024 Nov 4;4(11):e0003421. doi: 10.1371/journal.pgph.0003421 (PMC11534246; doi:10.1371/journal.pgph.0003421)
Supplement: S1 Appendix — (DOCX) [file pgph.0003421.s002.docx]

**Recovery from Ebola in Sierra Leone**

***English Questionnaire***

**DATE OF INTERVIEW** [__|__][__|__] (DAY, MONTH)

**TIME STARTED** [__|__][__|__] (24 HOUR TIME)

**INTERVIEWER NAME** [__________________________]

**INTERVIEWER NUMBER** [__|__|__]

**Characteristics of the Respondent**

**C1 Is this person an Ebola survivor?**

**C2 Does this person live in urban, town or rural area?** 0=Rural, 1=Town, 2=Urban

**C3 Is the respondent Male or Female**  0=Female, 1=Male

**1 DEMOGRAPHICS**

| **D1 How old were you at your last birthday?** | \| Age \|  \| \| --- \| --- \| |
| --- | --- | --- | --- |
| **D2 What is the highest level of school you attended: primary, secondary, or higher?** | NONE…………………………………….0  PRIMARY . . . . . . . . . . . . . . . . . . . . . . 1  JUNIOR SECONDARY . . . . . . . . . . . . . . 2  SENIOR SECONDARY . . . . . . . . . . . . . . 3  VOCATIONAL / COMMERCIAL / NURSING  TECHNICAL / TEACHING . . . . . . . 4  UNIVERSITY . . . . . . . . . . . . . . . . . . . . . . 5 |
| **D3 What is the highest (grade / form / year) you completed at that level?**  IF COMPLETED LESS THAN ONE YEAR AT THAT LEVEL, RECORD '00'. | GRADE / FORM / YEAR ______________ |

| **D4 What ethnic group do you belong to?**  (DO NOT READ LIST, ONLY ONE ANSWER IS POSSIBLE) | KRIO . . . . . . . . . . . . . ……….. . . . . . . . . . . 1  FULLAH . . . . . . . . . . . ……. . . . . . . . . . . . . 2  KISSI . . . . . . . . . . . . ……… . . . . . . . . . . . . 3  KONO . . . . . . . . . . . …… . . . . . . . . . . . . . . 4  LIMBA . . . . . . . . . . . …… . . . . . . . . . . . . . . 5  LOKO . . . . . . . . . . . . …… . . . . . . . . . . . . . 6  MANDINGO . . . . . . . . . ….. . . . . . . . . . . . . 7  MENDE. . . . . . . . . . . . . . …. . . . . . . . . . . . 8  SHERBRO . . . . . . . . . . .. ….. . . . . . . . . . . 9  TEMNE. . . . . . . . . . . . . . …. . . . . . . . . . . . 10  OTHER SIERRA LEONE (specify)…..95 (D4.95)  OTHER FOREIGN (specify)_____________96 (D4.96) |
| --- | --- |
| **D5 What religion are you?**  INTERVIEWER: CIRCLE CODE FOR RELIGION | No religion 0  Catholic 1  Muslim 3  Presbyterian 4  Baptist 5  Anglican 6  Pentecostal 7  Seventh Day Adventist 8  Jehovah’s Witnesses 9  Church of Christ 10  Indigenous Christian / AIC 11  Other (SPECIFY____________)….12 (D5.12) |
| **D6 When was the last time you went to a church/mosque?** | In the last week 1 in the last month 2 Last 2-6 Months 3 6 months or more 4 Never 5 Don’t know 6 |
| **D7 Please say how much you agree or disagree with the following statement)**  *The events in my life unfold according to a divine or greater plan*  **READ ALL RESPONSES** | Strongly disagree ..........................................1  Somewhat disagree…………………………2  Neither agree nor disagree……………….3  Somewhat agree…………………………….4  Strongly agree………………………………5 |
| **D8 Was anyone in your household infected with Ebola?** | Yes…………………………………………….1  No……………………………………………...0 |
| **D9 What was their relation to you?** | Grandparent…………………………1 (D9.1)  Parent…………………………………2 (D9.2)  Sibling…………………………………3 (D9.3)  Aunt/Uncle……………………………4 (D9.4)  Niece/Nephew…………………………5 (D9.5)  Cousin…………………………………..6 (D9.6)  Other (specify) ……………………..…7 (D9.7) |

**2 ECONOMIC STATUS**

**The following questions are to know about your economic status before and after Ebola. We are not trying to learn about your economic needs so that we can meet them, we are instead interested in how your economic status has changed since the Ebola epidemic.**

| \| **E1** \| **Does your household own any of the following (E1.1)** \| \| **Did your household own any of the following seven years ago**  **(before the 2014 Ebola epidemic)?**  **Note: it doesn’t have to be the exact same item, just the same type (E1.2)** \| \| \| --- \| --- \| --- \| --- \| --- \| \|  \| \|  \| No \| Yes \| No \| Yes \| \| A Bed with mattress \| 0 \| 1 \| 0 \| 1 \| \| B Sofa Set \| 0 \| 1 \| 0 \| 1 \| \| C Table and Chair(s) \| 0 \| 1 \| 0 \| 1 \| \| E Television \| 0 \| 1 \| 0 \| 1 \| \| F Radio \| 0 \| 1 \| 0 \| 1 \| \| G Mobile phone \| 0 \| 1 \| 0 \| 1 \| \| H Non mobile phone \| 0 \| 1 \| 0 \| 1 \| \| I Solar electricity panels \| 0 \| 1 \| 0 \| 1 \| \| J Computer \| 0 \| 1 \| 0 \| 1 \| \| K Motorcycle \| 0 \| 1 \| 0 \| 1 \| \| N Power generator \| 0 \| 1 \| 0 \| 1 \| \| O Refrigerator \| 0 \| 1 \| 0 \| 1 \| \| P Electric Iron \| 0 \| 1 \| 0 \| 1 \| \| Q Electricity \| 0 \| 1 \| 0 \| 1 \| \| R Metal roof \| 0 \| 1 \| 0 \| 1 \| |
| --- | --- | --- | --- | --- | --- | --- | --- | --- | --- | --- | --- | --- | --- | --- | --- | --- | --- | --- | --- | --- | --- | --- | --- | --- | --- | --- | --- | --- | --- | --- | --- | --- | --- | --- | --- | --- | --- | --- | --- | --- | --- | --- | --- | --- | --- | --- | --- | --- | --- | --- | --- | --- | --- | --- | --- | --- | --- | --- | --- | --- | --- | --- | --- | --- | --- | --- | --- | --- | --- | --- | --- | --- | --- | --- | --- | --- | --- | --- | --- | --- | --- | --- | --- | --- | --- | --- |

**3 FERTILITY, CHILDBEARING, AND CONTRACEPTION**

**Now I’d like to ask you some questions about childbearing and contraceptive use:**

| **F1** | **Women: Now let’s talk about your own children. Can you give me the total number of children you have ever given birth to? (F1.1)**  **Men:**  **Now let’s talk about your own children. Can you give me the total number of children you have fathered? (F1.2)** | Number [________]  if respondent is childless write “0”  **if none, write “0”** |  |
| --- | --- | --- | --- |
| **F2** | **Would you please tell me the number of male and female children you gave birth to who are still living?** | Males alive [________] (F2.1)  Females alive [________] (F2.2)  **if none, write “0”** |  |

**4 MARRIAGE**

**Now I’d like to ask you some questions about your marriage history:**

| **M1 Are you now married or living with a spouse/partner, or are you now widowed, divorced, or no longer living together?** | Married/living together 1  Separated 2  Divorced 3  Widowed 4  Never married 5 **🡪 COV_1** |
| --- | --- |

**6 HEALTH**

| **H1: Signs/Symptoms**  **Select all of the signs and symptoms that are present now.** | **H2 For how many years has this been a problem for you?** | **H4 Rate how much this problem interferes now in your life.**  **1 Does not interfere at all**  **2 Interferes small**  **3 Interferes a lot** |
| --- | --- | --- |
| **A Weight Loss** |  |  |
| **B Fatigue/weakness** |  |  |
| **C Headaches** |  |  |
| **D Confusion** |  |  |
| **E Appetite Loss** |  |  |
| **F Vision Problems** |  |  |
| **G Hearing loss** |  |  |
| **H Heart problems** |  |  |
| **I Difficulty breathing** |  |  |
| **J Chest pain** |  |  |
| **K Stomach pain** |  |  |
| **L Diarrhea** |  |  |
| **M Decreased libido** |  |  |
| **N Joint pain** |  |  |
| **O Numbness of extremities** |  |  |
| **P Feeling suddenly scared for no reason** |  |  |
| **Q Loss of sexual interest or pleasure** |  |  |
| **R Difficulty falling asleep or staying asleep** |  |  |
| **S Feeling fearful** |  |  |
| **T Spells of terror or panic** |  |  |
| **U Trembling (Tremors)** |  |  |

**11 QUESTIONS FOR THE INTERVIEWER**

**SOON AFTER THE INTERVIEW, PLEASE ANSWER THE FOLLOWING QUESTIONS**

| I1 Ability of respondent to understand and respond to questions? | Answered most questions with ease 1  Did not understand some of the questions and answer choices 2  Did not understand most questions and answer choices 3 | |
| --- | --- | --- |
| I2 Degree of cooperation  **Note to Interviewer:** circle the degree of cooperation compared to other respondents, not overall degree of cooperation | Very uncooperative 1  Uncooperative 2  Average 3  Cooperative 4  Very Cooperative 5 | |
| I3 How well acquainted are you with the respondent? | Very well acquainted 1  Well acquainted 2  Not well acquainted 3  Not acquainted 4 | |
| I4 Enter Interviewer ID: | | ID: [___\|___] |

**End of Survey**

**TIME FINISHED** [__|__][__|__] (24 HOUR TIME)
