# Supplementary material for: Long-term physical and mental health outcomes of Ebola Virus Disease survivors in Kenema District, Sierra Leone: A cross-sectional survey
Source: PLOS Glob Public Health. 2024 Nov 4;4(11):e0003421. doi: 10.1371/journal.pgph.0003421 (PMC11534246; doi:10.1371/journal.pgph.0003421)
Supplement: Attachment — Submitted filename: PGPH-D-24-00262_reviewer.doc [file pgph.0003421.s004.doc]

**PLOS Global Public Health**

**Long-term physical and mental health outcomes of Ebola Virus Disease survivors in Kenema District, Sierra Leone: a cross-sectional survey**

|  | --Manuscript Draft-- |
| --- | --- |
|  |  |
| **Manuscript Number:** | PGPH-D-24-00262 |
| **Article Type:** | Research Article |
|  |  |
| **Full Title:** | Long-term physical and mental health outcomes of Ebola Virus Disease survivors in |
|  | Kenema District, Sierra Leone: a cross-sectional survey |
| **Short Title:** | Long-term physical and mental health outcomes of Ebola Virus Disease survivors |
|  |  |
| **Corresponding Author:** | Jenna Nicole Bates, MSc |
|  | University of Copenhagen School of Global Health: Kobenhavns Universitet School of |
|  | Global Health |
|  | Copenhagen, DENMARK |
| **Order of Authors:** | Jenna N. Bates, MSc |
|  |  |
|  | Abdulai Kamara, BSc |
|  |  |
|  | Mohamed Sheku Bereteh, BSc |
|  |  |
|  | Denise Barrera, MPH, MSW |
|  |  |
|  | Lina Moses, PhD, MSPH |
|  |  |
|  | Allieu Sheriff |
|  |  |
|  | Fudia Sesay |
|  |  |
|  | Mohamed S. Yillah |
|  |  |
|  | Donald Grant, MBChB, MPH |
|  |  |
|  | Joseph Lamin, MPH |
|  |  |
|  | Philip Anglewicz, PhD |
|  |  |
| **Keywords:** | Ebola survivors; mental health; physical health; Ebola virus disease; long-term |
|  | impacts of epidemics |
| **Abstract:** | The 2014-2016 Ebola Virus Disease (EVD) epidemic in West Africa was the deadliest |
|  | in history, with over 14,000 cases. Numerous physical and mental health symptoms |
|  | have been reported in EVD survivors, although there is limited prior research on long- |
|  | term health issues, and how the health of survivors compares to the general the |
|  | population. We conducted a population-based survey of EVD survivors in Kenema |
|  | District, Sierra Leone and a sample of community members, and compared resulting |
|  | data about self-reported symptoms, duration, and severity between EVD survivors and |
|  | community members through multivariate regression models. This study found that 6 |
|  | years after the epidemic, survivors were significantly more likely to experience both |
|  | physical and mental health symptoms than community members, with respective AORs |
|  | of 2.79 (95% CI, 2.42-3.21), p < 0.001, and 13.59 (95% CI, 8.15-22.67), p < 0.001. The |
|  | most reported physical health symptoms exhibited by EVD survivors were joint pain |
|  | (75.5%), headaches (68.2%), and vision problems (44.5%), and the most prevalent |
|  | psychological symptoms were spells of terror and panic (25.5%) and difficulty falling |
|  | asleep or staying asleep (20.0%). EVD survivors were significantly more likely than |
|  | community members to report the symptoms as lasting for a longer period (an average |
|  | of 5.18[[EQUATION]]2.34 years) and with higher severity. The results indicated that six |
|  | years after the epidemic, EVD survivors in Kenema District, Sierra Leone are |
|  | experiencing worse physical and mental health than their peers. These findings of the |
|  | long-term, debilitating health issues following EVD infection should be considered |
|  | when designing and implementing future epidemic responses. |
|  |  |
| **Additional Information:** |  |
| **Question** | **Response** |
|  |  |

Powered by Editorial Manager® and ProduXion Manager® from Aries Systems Corporation

| **Financial Disclosure** | | | | | | | This work was funded by the NIH under grant number 1R21HD098504: The Impact of |
| --- | --- | --- | --- | --- | --- | --- | --- |
|  |  |  |  |  |  |  | Ebola Infection on Demographic and Social Outcomes in Sierra Leone, received by PA |
|  |  |  |  |  |  |  | and LM. The funders had no role in study design, data collection and analysis, decision |
|  |  |  |  |  |  |  | to publish, or preparation of the manuscript. |
| Enter a financial disclosure statement that | | | | | | |  |
| describes the sources of funding for the | | | | | | |  |
| work included in this submission and the | | | | | | |  |
| role the funder(s) played. This includes | | | | | | |  |
| grants and any commercial funding of the | | | | | | |  |
| work or authors. | | | | | | |  |
| This statement will be typeset if the | | | | | | |  |
| manuscript is accepted for publication. | | | | | | |  |
| *Please review the [submission guidelines](http://journals.plos.org/globalpublichealth/s/submission-guidelines#loc-financial-disclosure-statement)* | | | | | | |  |
|  |  |  |  |  |  |  |  |
| *and the instructions link below for detailed* | | | | | | |  |
| *requirements and guidance.* | | | | | | |  |
| **Competing Interests** | | | | | | | No competing interests exist. |
| On behalf of all authors, disclose any | | | | | | |  |
| competing interests that could be | | | | | | |  |
| perceived to bias this work. | | | | | | |  |
| This statement will be typeset if the | | | | | | |  |
| manuscript is accepted for publication. | | | | | | |  |
| *Please review the instructions link below* | | | | | | |  |
| *and PLOS Global Public Health’s* | | | | | | |  |
| [*competing interests*](https://journals.plos.org/globalpublichealth/s/competing-interests) *policy to determine* | | | | | | |  |
|  |  | |  |  |  | |  |
| *what information must be disclosed at* | | | | | | |  |
| *submission.* | | | | | | |  |
| **Data Availability** | | | | | | | If the paper is accepted, data will be deposited in an acceptable public repository prior |
|  |  |  |  |  |  |  | to submission. |
| Before publication, Authors are required | | | | | | |  |
| to make fully available and without | | | | | | |  |
| restriction all data underlying their | | | | | | |  |
| findings. Please see our [PLOS Data](https://journals.plos.org/globalpublichealth/s/data-availability) | | | | | | |  |
|  |  | | |  |  | |  |
| [Policy](https://journals.plos.org/globalpublichealth/s/data-availability) page for detailed information on | | | | | | |  |
|  |  | | | | | |  |
| this policy. | | | | | | |  |
| A **Data Availability Statement**, detailing | | | | | | |  |
| where the data can be accessed, is | | | | | | |  |
| required at first submission. Insert your | | | | | | |  |
|  |  |  |  |  |  |  |  |

*Powered by Editorial Manager® and ProduXion Manager® from Aries Systems Corporation*

Data Availability Statement in the box

below.

Please see the [data reporting](https://journals.plos.org/globalpublichealth/s/submission-guidelines#loc-data-reporting) section of

our submission guidelines for instructions

on what you need to include in your Data

Availability Statement.

This statement will be typeset if the

manuscript is accepted for publication.

*PLOS allows rare exemptions to address*

*legal and ethical concerns. If you have*

*legal or ethical restrictions, please use the*

*box below to detail these in full sentences*

*for the Journal team to consider.*

*Powered by Editorial Manager® and ProduXion Manager® from Aries Systems Corporation*

Manuscript

[Click here to access/download;Manuscript;EVD Survivor](https://www.editorialmanager.com/pgph/download.aspx?id=300804&guid=724f349d-31b6-4b94-990f-b39ad82c64c2&scheme=1) [Health Outcomes.docx](https://www.editorialmanager.com/pgph/download.aspx?id=300804&guid=724f349d-31b6-4b94-990f-b39ad82c64c2&scheme=1)

1 **Long-term physical and mental health outcomes of Ebola**
2 **Virus Disease survivors in Kenema District, Sierra Leone: a**
3 **cross-sectional survey**
4 **Authors**
5 Jenna N. Bates,^1*^ Abdulai Kamara,^2^ Mohamed Sheku Bereteh,^2^ Denise Barrera,^3^ Lina
6 Moses,^3^ Allieu Sheriff,^4^ Fudia Sesay,^4^ Mohamed S. Yillah,^4^ Donald S. Grant,^5,6^ Joseph
7 Lamin,^2^ Philip Anglewicz^7*^

| 8 |  |  |
| --- | --- | --- |
| 9 | *^1^ University of Copenhagen, School of Global Health* | |
| 10 | *2* | *Njala University, School of Community Health Sciences* |
| 11 | *3* | *Tulane University, School of Public Health and Tropical Medicine* |
| 12 | *4* | *Sierra Leone Association of Ebola Survivors* |
| 13 | *5* | *Kenema Government Hospital* |
| 14 | *6* | *College of Medicine and Allied Health Sciences, University of Sierra Leone* |
| 15 | *7* | *Johns Hopkins Bloomberg School of Public Health* |
| 16 |  |  |

17 **Corresponding author*
18 *E-mail:* [*jennab318@gmail.com*](mailto:jennab318@gmail.com)

19

20 **Corresponding author*
21 *E-mail:* [*panglew@jhu.edu*](mailto:panglew@jhu.edu)

1

22 **Abstract**

23	The 2014-2016 Ebola Virus Disease (EVD) epidemic in West Africa was the

24 deadliest in history, with over 14,000 cases. Numerous physical and mental health symptoms
25 have been reported in EVD survivors, although there is limited prior research on long-term
26 health issues, and how the health of survivors compares to the general population. We
27 conducted a population-based survey of EVD survivors in Kenema District, Sierra Leone and
28 a sample of community members, and compared resulting data about self-reported symptoms,
29 duration, and severity between EVD survivors and community members through multivariate
30 regression models. This study found that 6 years after the epidemic, survivors were
31 significantly more likely to experience both physical and mental health symptoms than
32 community members, with respective AORs of 2.79 (95% CI, 2.42-3.21), *p* < 0.001, and
33 13.59 (95% CI, 8.15-22.67), *p* < 0.001. The most reported physical health symptoms
34 exhibited by EVD survivors were joint pain (75.5%), headaches (68.2%), and vision
35 problems (44.5%), and the most prevalent psychological symptoms were spells of terror and
36 panic (25.5%) and difficulty falling asleep or staying asleep (20.0%). EVD survivors were
37 significantly more likely than community members to report the symptoms as lasting for a
38 longer period (an average of 5.18±2.34 years) and with higher severity. The results indicated
39 that six years after the epidemic, EVD survivors in Kenema District, Sierra Leone are
40 experiencing worse physical and mental health than their peers. These findings of the long-
41 term, debilitating health issues following EVD infection should be considered when
42 designing and implementing future epidemic responses.
43 **Introduction**

44	Despite the prominence of Ebola Virus Disease (EVD), little is known about its long-

45 term impact on physical and mental health. EVD is a severe hemorrhagic fever that has 2
46 impacted tens of thousands of individuals since its discovery in the Democratic Republic of
47 Congo (DRC) in 1976. The 2014-2016 EVD epidemic in West Africa was the deadliest in
48 history, with 28,652 suspected, probable, or confirmed cases [1]. It was largely centered in
49 Liberia, Guinea, and Sierra Leone, but had a far reach with limited, quickly controlled cases in
50 Nigeria, Mali, Senegal, the United States, the United Kingdom, and Spain. Sierra Leone was
51 the most heavily impacted by the epidemic, with a reported 14,124 cases, 3,956 deaths and
52 more suspected [1]. With a case fatality rate of 40% and a tendency to spread in intimate
53 interpersonal activities such as caregiving and burial processes, EVD was highly destructive to
54 communities and households throughout the affected regions [2].

55	“Post-Ebola Syndrome” is used to describe the physical sequelae experienced by many

56 survivors, with joint and muscle pain, fever, headaches, and ocular symptoms being among the
57 most commonly reported sequelae [3–5]. Female EVD survivors have also experienced
58 reproductive health sequelae, such as irregular menstruation, infertility, spontaneous abortions,
59 and stillbirths [6].

60	There are also several studies concerning the mental health of survivors, finding that

61 they are at higher risk of developing depression, anxiety, and post-traumatic stress disorder
62 (PTSD) [7–13]. This is consistent with findings from other epidemics and disaster scenarios,
63 in which experiences during and after the event such as hospitalization, isolation, loss, having
64 a life-threatening illness, and subsequent stigmatization, can all contribute to psychosocial
65 distress [14]. The reported rates of mental illness in EVD survivors varies, with a systematic
66 review by Lötsch et al. (2017) reporting prevalence of depression in EVD survivors ranging
67 from 17 to 50% [9]. In addition, reported rates are often incomparable due to variations in the
68 scales or diagnostic methods utilized. EVD survivors have also faced negative social,
69 demographic, and economic outcomes, with prior research finding that survivors have

3

| 70 | experienced stigma, rejection by their community post-hospital discharge, permanent |
| --- | --- |
| 71 | displacement, economic losses, and dissolution of their marriages [2,8,11,12,15,16]. |
| 72 | Although previous research has documented the impacts of EVD upon the physical and |

73 mental health of survivors, these studies have important limitations. The vast majority of
74 research on the impacts of Ebola infection is qualitative and used convenience samples
75 [6,11,12,17–28]. As a result, these studies are vulnerable to selection bias and the extent to
76 which the results apply to a broader population is unknown. In addition, much of the
77 quantitative research on the social and demographic impacts of Ebola was conducted with small
78 samples of less than 120 participants, which is therefore vulnerable to type two errors. Few
79 studies included a comparison group of uninfected individuals, which is necessary to accurately
80 identify the impact of Ebola infection on various outcomes. In addition, although many believe
81 that the impact of Ebola is long-lasting, research has only examined the impacts of Ebola
82 infection occurring shortly after recovery. As a result, despite the widespread knowledge of
83 EVD, little is known about the long-term physical and mental health impacts on EVD
84 survivors.
85 We address the limitations of previous research here. To do so, we use population-based
86 data from Eastern Province of Sierra Leone, which includes both EVD survivors and a
87 comparison group. These data were collected October - November 2021, approximately five
88 to seven years after the outbreak in this region. Our goal was to identify the long-term impact
89 of EVD on physical and mental health outcomes on survivors.
90 **Methods**
91 **Setting**
92 The 2014-2016 West Africa epidemic began in the Mano River triangle at the intersection
93 of Guinea, Liberia, and Sierra Leone, inhabited by the cross-border Kissi tribe [29]. The first

4

94 case of EVD, the spillover event, was likely a small boy who had been playing in a tree
95 inhabited by bats in Meliandou, Guinea [2,29]. Due to the fluidity of family borders and the
96 ease of crossing borders by road, foot, and boat, the virus quickly spread to Sierra Leone and
97 Liberia [29]. In Sierra Leone, EVD cases were hidden for months after its arrival, in part due
98 to mistrust and fear, emerging only after the virus was already propagating in Kailahun and
99 Kenema Districts [29].
100 The Eastern Province, including Kenema District, served as an early epicenter of the
101 outbreak in Sierra Leone. Many health care workers contracted EVD [29], followed by their
102 family members, and the community at large, with a lab-confirmed case count of 407 and more
103 suspected in Kenema District [30,31]. Cases in Kenema spanned from mid-2014 to 2015 before
104 lulling to a stop, largely due to increased isolation measures and use of ETUs by affected
105 communities [29].
106 **Sampling and recruitment**

| 107 | All study participants were recruited and surveyed from October 23^rd^ – November 23^rd^, |
| --- | --- |
| 108 | 2021. |
| 109 | In this study, EVD survivors were identified from the national registry of Ebola |

110 infections maintained by our partner, the Sierra Leone Association of Ebola Survivors
111 (SLAES). This registry was created during the epidemic and lists all confirmed Ebola cases in
112 Eastern Province of Sierra Leone, along with some characteristics, including age, sex, area of
113 residence when infected, whether they died from infection, and full contact information
114 (including location and phone number, if available). At the time of data collection, there were
115 167 EVD survivors in the SLAES-Kenema database, making it one of the most affected
116 districts.

5

117	EVD survivors were recruited by SLAES-Kenema executive board members. The

118 entire population (n = ????) of EVD survivors over 15 years of age in Kenema District was contacted,
119 and the resulting sample was an estimated 76.9% of eligible EVD survivors (110 out of 143).
120 Control group participants were selected via a systematic random sample of 40
121 households within eight enumeration areas (EAs), a total of 320 households. EAs were selected
122 by probability sampling weighted by the number of survivors in each area, determined by
123 aggregating the SLAES survivor database by street name and section (the equivalent of a
124 neighborhood). The sampling frame for each EA was selected based on the population size,
125 which was obtained from the Statistics Sierra Leone 2020 census. Eligible households were
126 selected randomly, as was one participant per household.

127	Participants were excluded from the study if they were under 15 years of age or if they

128 did not live in Kenema District. EVD survivors were also excluded if they were not in the
129 SLAES database. Otherwise, all EVD survivors and community members selected via the
130 sampling frame were eligible to participate in the survey.
131 **Data collection**

132	Participants were interviewed by twelve field researchers from Njala University,

133 supervised by two members of the Kenema Government Hospital Outreach Team.
134 Interviewers and supervisors attended a one-week training prior to data collection where they
135 were taught the protocol and practiced interviewing participants. All interviews were
136 conducted verbally in Krio or Mende due to low literacy rates in the target population. The
137 survey was run on smart phones using the Open Data Collect app, and data was uploaded to
138 the ONA server.
139 **Measures**

140	The survey instrument consists of sections concerning demographics, economics,

141 fertility and contraception, marital history, sexual behavior, health, health care access, social 6
142 capital, community, stigma, migration, and COVID-19. Physical and mental health sequalae
143 were evaluated with a checklist consisting of signs and symptoms prevalent in EVD
144 survivors, adapted from the literature.
145 **Data analysis**

146	De-identified data was analyzed in SPSS Version 28, using a variety of summary

147 statistics, comparison of means tests, and bivariate and multivariate regressions based on the
148 nature of the questions and the dependent variables.
149 **Participant demographics**

150	Demographic variables were analyzed for frequency of responses, and then for

151 significant differences between the EVD survivor and community member groups through chi
152 square analyses (gender, education, location marital status, ethnicity, religion, wealth status),
153 or t-tests (age, number of children), depending upon the nature of the dependent variable
154 (categorical or continuous, respectively).

155	Wealth was measured via proxy indicators due to the difficulties of collecting

156 comparable data about the long-term economic statuses of households. Instead, participants
157 were asked about their household assets, for example, ownership of electricity or a radio. These
158 variables were utilized to generate Wealth Indices through a Principal Components Analysis,
159 with a varimax rotation, extraction using eigenvalues greater than 1, and a maximum of 25
160 iterations for convergence [33]. Cases were then ranked into equivalent tertiles, in a method
161 adapted from programs such as the Demographic Health Survey and World Food Program
162 [33,34]. This measure of wealth is relative to the rest of the dataset, categorizing participants
163 with “higher,” “middle,” or “lower” wealth statuses.

164

7

165 **Analytic methods**

166	Total symptom counts and separate physical and mental health symptom counts were

167 analyzed with Poisson regression models. Symptoms that had arisen before the EVD outbreak
168 began, eight years prior to the survey, were excluded. A Poisson regression model was selected
169 due to the right skew of the dependent variable, determined by running the Kolmogorov-
170 Smirnov and Shapiro-Wilk tests for normality, with significances of <0.001 for the EVD
171 survivor and community member groups. A Poisson regression adequately fit the data, as the
172 dependent variable consisted of count data, the independent variable and covariates were
173 categorical or continuous, observations were independent, and count data generally followed a
174 Poisson distribution with slight overdispersion, assessed with the Pearson dispersion statistic.
175 Health signs/symptoms questions were analyzed via binomial logistic regression
176 models due to the binary nature of the independent variable of Ebola survivorship (*0 or 1*) and
177 the dependent variable of the presence of each symptom (*0 or 1*). Symptoms that had arisen
178 before the EVD outbreak began, eight years prior to the survey, were excluded. Data consisted
179 of independent observations, with no multicollinearity of independent variables, no extreme
180 outliers, a linear relationship between independent variables and the logit of the response
181 variable, and a sufficiently large sample size.
182 Data regarding duration of symptoms and severity were obtained for each “yes”
183 response for symptom presence. Duration of symptoms was analyzed with descriptive outputs
184 and an independent t-test. Total ranking of symptom severity was analyzed with an ordinal
185 regression due to the ordered Likert scale of responses (no/little/a lot of interference).
186 Frequency outputs were run on total symptom interference ratings.

187	All multivariate models were adjusted for age, gender, and proxy indicators of

188 socioeconomic status (SES) including wealth status, education level, marital status, and
189 number of children still living due to prior association of the variables with the exposure of 8
190 EVD and the outcomes of physical and mental statuses [35] [36,37] [38,39] [40] [41] [42].
191 Location would also be included as a confounder due to its impacts upon EBOV infection
192 and health status [43], but it was not due to measurement error (see section 4.1).
193 **Ethical compliance**

194	Ethical clearance was obtained by the Institutional Review Boards of Johns Hopkins

195 University and Tulane University, the Sierra Leone Ethics Review Board, and the School of
196 Global Health of University of Copenhagen. Data was collected and stored in accordance with
197 the Principles of Helsinki and General Data Protection Regulation (GDPR) [44,45].

198	Verbal informed consent was obtained from each study participant, in their own

199 language (Krio or Mende). Verbal consent was utilized instead of written consent due to low
200 literacy rates amongst the target population. Participants from 15 to 18 years of age provided
201 assent, and one parent was required to provide consent for study participation. In accordance
202 with local customs, consent was also obtained from the SLAES-Kenema executive board and
203 the chiefs of entered communities.

204	Data was pseudo-anonymized. Coordinating identifiers were removed before analyzing

205 the data, in accordance with GDPR. All data was stored on secure servers. Anonymized survey
206 results were disseminated in forums of interested participants and study personnel, as well as
207 stakeholders at the local, national, and international levels.
208 **Results**
209 **Participant demographics**

210	Differences between demographics of the EVD survivor and community member

211 participant groups were calculated with independent t-tests and chi square tests, depending on
212 the nature of the dependent variable. T-tests were run to compare the means of age and number

9

213 of children due to their continuous nature, whereas chi square tests were run for categorical
214 dependent variables.
215 **Table 1. Demographics of Survey Participants.**

|  | **Participant Demographics** | **EVD Survivors** | | **Community** | | ***p-*Value** |
| --- | --- | --- | --- | --- | --- | --- |
|  |  | **(*n/N*)** | | **Members (*n/N*)** | |  |
|  | **Gender** |  |  |  |  | 0.328 |
|  | Female | 66.4% | (73/110) | 61.1% | (195/319) |  |
|  | Male | 33.6% | (37/110) | 38.9% | (124/319) |  |
|  | **Age (mean** ± **SD)** | 35.4 ± 14.4 | | 34.6 ±15.9 | | 0.604 |
|  | 15-19 | 11.8% | (13/110) | 11.9% | (38/319) |  |
|  | 20-29 | 25.5% | (28/110) | 37.9% | (121/319) |  |
|  | 30-39 | 27.3% | (30/110) | 20.4% | (65/319) |  |
|  | 40-49 | 20.9% | (23/110) | 11.0% | (35/319) |  |
|  | 50-59 | 10.0% | (11/110) | 8.8% | (28/319) |  |
|  | 60-69 | 0.9% | (1/110) | 5.3% | (17/319) |  |
|  | 70-79 | 0.9% | (1/110) | 3.4% | (11/319) |  |
|  | > 80 | 2.7% | (3/110) | 1.3% | (4/319) |  |
|  | **Marital Status** |  |  |  |  | <0.001^a^ |
|  | Married/living together | 44.5% | (49/110) | 49.8% | (159/319) |  |
|  | Separated/divorced | 5.5% | (6/110) | 6.0% | (19/319) |  |
|  | Widowed | 23.6% | (26/110) | 4.7% | (15/319) |  |
|  | Never married | 26.4% | (29/110) | 39.5 (126/319) | |  |
|  | **Number of Children (mean** ± **SD)** | 3.7 ± 3.3 | | 2.7 ± 3.0 | | 0.003^a^ |
|  | 0-3 | 51.8% | (57/110) | 69.0% | (220/319) |  |
|  | 4-7 | 38.2% | (42/110) | 24.4% | (78/319) |  |
|  | 8-11 | 7.3% | (8/110) | 4.7% | (15/319) |  |
|  | > 11 | 2.7% | (3/110) | 1.9% | (6/319) |  |
|  | **Location** |  |  |  |  | <0.001^a^ |
|  | Rural | 66.4% | (73/110) | 20.4% | (65/319) |  |
|  | Town | 5.5% | (6/110) | 38.9% (124/319 | |  |
|  | City | 28.2% | (31/110) | 40.8% | (130/319) |  |
|  | **Education Level** | 0.174 | |  |  |  |
|  | None | 43.6% | (48/110) | 32.0% | (102/319) |  |
|  | Primary | 8.2% | (9/110) | 9.1% | (29/319) |  |
|  | Junior Secondary | 9.1% (10/110) | | 9.7% | (31/319) |  |
|  | Senior Secondary | 23.6% | (26/110) | 34.5% | (110/319) |  |
|  | Higher | 15.5% | (17/110) | 14.6% | (47/319) |  |
|  | **Current Wealth Status** |  |  |  |  | 0.006^a^ |
|  | Lowest | 41.8% | (46/110) | 33.2% | (106/319) |  |
|  | Middle | 27.3% | (41/110) | 29.3% | (93/319) |  |
|  | Highest | 20.9% | (23/110) | 37.6% | (120/319) |  |
|  | **Ethnicity** | 0.466 | |  |  |  |
|  | Mende | 76.4% | (84/110) | 68.3% | (218/319) |  |
|  | Mandingo | 5.5% | (6/110) | 6.6% | (21/319) |  |
|  | Krio | 0.9% | (1/110) | 0.0% | (0/319) |  |
|  | Fullah | 3.6% | (4/110) | 4.1% | (13/319) |  |
|  | Kissi | 2.7% | (3/110) | 3.8% | (12/319) |  |
|  | Kono | 1.8% | (2/110) | 2.5% | (8/319) |  |
|  | Sherbro | 2.7% | (3/110) | 1.6% | (5/319) |  |
|  | Temne | 4.5% | (5/110) | 5.6% | (18/319) |  |
|  | Other Sierra Leone | 1.8% | (2/110) | 2.5% | (8/319) |  |
|  | Limba | 0.0% | (0/110) | 4.1% | (13/319) |  |
|  | Loko | 0.0% | (0/110) | 0.3% | (1/319) |  |
|  | Other Foreign | 0.0% | (0/110) | 0.6% | (2/319) |  |
|  | **Religious Affiliation** |  |  |  |  | 0.338 |
|  | Muslim | 73.6% | (81/110) | 69.3% | (221/319) |  |

10

|  |  | Christian | 26.3% (29/110) | 30.7% (98/319) |
| --- | --- | --- | --- | --- |
| 216 | ^a^Statistically significant (p < 0.05). | |  |  |
| 217 |  | Table 1 shows that the demographic compositions of the EVD survivor sample and the | | |

218 community member-based comparison group were relatively similar but demonstrated
219 significant differences in marital status, number of children, location, and wealth status.
220 Survivors had more children on average (*p* = 0.003), had less wealth (*p* = 0.004), and lived in
221 more rural areas (*p* < 0.001). In addition, they were more likely to be widowed, at 23.6% vs.
222 4.7% (*p* < 0.001). These differentials reinforce the need to adjust for confounders.

223	In both respondent groups, females were slightly more represented than men, at nearly

224 a 1:2 ratio. Both respondent groups were Mende and Muslim by majority. Represented ages
225 skewed younger and older in the comparison group, with a greater number of respondents in
226 the 20-29 and 60-79 age ranges, and less in the 30-59 age ranges. While not significantly
227 different, education levels also varied, with EVD survivors more likely to have no education
228 (43.6%) than community members (32.0%).

229	Differences in ages and genders may reflect limitations of the sampling methodology.

230 Due to the survey data collection in the harvest period at the beginning of the dry season
231 (October-November 2021) [46], middle-aged men may have been working at their farms, while
232 younger and older individuals (primarily female) may have been at home. Gender differentials
233 may also match the demographics of the EVD survivor sample due to similar gender norms, as
234 females are thought to be more likely to be infected with EBOV due to their traditional role as
235 caretakers [35].

236	Variances in location may be explained by the sampling methodology or due to

237 measurement error. 279 out of 319 (87.5%) of community members were sampled from
238 communities within Kenema Town, which is primarily an urban area. It is likely that more
239 EVD survivors live in rural areas, with at least 18.2% of eligible survivors living outside of
240 Nongowa Chiefdom (where Kenema Town is situated) and more living in less urban areas of 11

241 Nongowa. However, there are likely measurement errors due to interviewer misunderstandings
242 of the answer options (urban, town, or rural) that were revealed during the data collection
243 period, as definitions of the answer options were unclear. Therefore, it cannot be used as a
244 reliable measure of location.
245 **Sequelae of Ebola survivors**

246	EVD survivors demonstrated significantly higher rates of physical and mental health

247 sequelae associated with post-Ebola syndrome. They were asked to select the signs and
248 symptoms that they were currently experiencing from a list. Signs and symptoms present >8
249 years, since before the beginning of the EVD epidemic, were excluded.

250	A Poisson regression was run to predict the total number of reported symptoms present

251 based on their survivor status and adjusted for confounders (age, gender, education, marital
252 status, number of children, and wealth status). EVD survivors are 3.22 (95% CI, 2.82-3.67)
253 times more likely to experience physical and mental health symptoms than members of the
254 comparison group, a statistically significant result at *p* <0.001. Unadjusted, the OR was 3.15
255 (95% CI, 2.78-3.57). In total, EVD survivors experience a mean of 4.69 ± 3.26 reported
256 sequelae, whereas the comparison group had a mean of 1.49 ± 1.28.

257	When viewed separately, groups of physical and mental health symptoms were both

258 more likely amongst EVD survivors than community members, with respective AORs of 2.79
259 (95% CI, 2.42-3.21), *p* < 0.001, and 13.59 (95% CI, 8.15-22.67), *p* < 0.001. Unadjusted ORs
260 were 2.75 (95% CI, 1.30-1.56) and 12.97 (95% CI, 7.89-21.33).
261 **Self-reported physical and mental health symptoms**

262	In addition, binomial logistic regressions were run to determine the comparative odds

263 of EVD survivors experiencing each sequela versus community members. Bivariate and
264 multivariate regressions adjusted for age, gender, education, marital status, number of children,
265 and wealth status were run to generate ORs and AORs. 12
266 **Table 2. Rates of Sequelae in EVD Survivors and Community Members.**

|  |  | **Signs/Symptoms** | **EVD** | **Community** | **Unadjusted** | **Adjusted^a^** | ***p*-Value** |
| --- | --- | --- | --- | --- | --- | --- | --- |
|  |  |  | **Survivors** | **Members** | **OR (95% CI)** | **OR (95% CI)** |  |
|  |  |  | **(*n*/*N*)** | **(*n*/*N)*** |  |  |  |
|  |  | **Physical Health Symptoms** | |  |  |  |  |
|  |  | Weight loss | 11.8% | 6.0% | 2.12 | 2.26 | 0.039^b^ |
|  |  |  | (13/110) | (19/319) | (1.01-4.44) | (1.04-4.91) |  |
|  |  | Fatigue/weakness | 31.8% | 17.2% | 2.51 | 2.53 | <0.001^b^ |
|  |  |  | (35/110) | (55/319) | (1.52-4.15) | (1.51-4.25) |  |
|  |  | Headaches | 68.2% | 47.6% | 2.63 | 2.86 | <0.001^b^ |
|  |  |  | (75/110) | (152/319) | (1.67-4.14) | (1.78-4.60) |  |
|  |  | Confusion | 24.5% | 7.8% | 4.19 | 4.32 | <0.001^b^ |
|  |  |  | (27/110) | (25/319) | (2.28-7.68) | (2.29-8.13) |  |
|  |  | Appetite loss | 17.3% | 9.1% | 2.26 | 2.27 | 0.016 ^b^ |
|  |  |  | (19/110) | (29/319) | (1.20-4.25) | (1.16-4.43) |  |
|  |  | Vision problems | 44.5% | 4.7% | 22.49 | 24.03 | <0.001^b^ |
|  |  |  | (49/110) | (15/319) | (11.07-45.71) | (11.41-50.61) |  |
|  |  | Hearing loss | 13.6% | 1.3% | 15.36 | 15.82 | <0.001^b^ |
|  |  |  | (15/110) | (4/319) | (4.32-54.57) | (4.33-57.82) |  |
|  |  | Heart problems | 26.4% | 4.1% | 12.33 | 10.70 | <0.001^b^ |
|  |  |  | (29/110) | (13/319) | (5.61-27.09) | (4.72-24.26) |  |
|  |  | Difficulty breathing | 8.2% | 1.6% | 5.60 | 7.60 | <0.001^b^ |
|  |  |  | (9/110) | (5/319) | (1.83-17.08) | (2.34-24.67) |  |
|  |  | Chest pain | 24.5% | 4.7% | 7.92 | 8.26 | <0.001^b^ |
|  |  |  | (27/110) | (15/319) | (3.83-16.35) | (3.85-17.72) |  |
|  |  | Stomach pain | 30.9% | 19.1% | 2.20 | 2.55 | <0.001^b^ |
|  |  |  | (34/110) | (61/319) | (1.33-3.64) | (1.49-4.38) |  |
|  |  | Diarrhea | 0.0% | 1.3% | 0.00 | 0.00 | 0.996 |
|  |  |  | (0/110) | (4/319) | (0.00-0.00) | (0.00-0.00) |  |
|  |  | Decreased libido | 4.5% | 0.9% | 5.02 | 6.11 | 0.019^b^ |
|  |  |  | (5/110) | (3/319) | (1.18-21.35) | (1.34-27.89) |  |
|  |  | Joint pain | 75.5% | 31.3% | 7.47 | 7.89 | <0.001^b^ |
|  |  |  | (83/110) | (110/319) | (4.54-12.28) | (4.68-13.30) |  |
|  |  | Numbness of | 13.6% | 1.3% | 12.43 | 10.62 | <0.001^b^ |
|  |  | extremities | (15/110) | (4/319) | (4.03-38.36) | (3.23-34.90) |  |
|  |  | **Mental Health Symptoms** | |  |  |  |  |
|  |  | Feeling suddenly | 13.6% | 0.3% | 50.21 | 64.27 | <0.001^b^ |
|  |  | scared for no reason | (15/110) | (1/319) | (6.55-385.06) | (8.08-511.03) |  |
|  |  | Loss of sexual | 14.5% | 1.9% | 8,88 | 8.92 | <0.001^b^ |
|  |  | interest or pleasure | (16/110) | (6/319) | (3.38-23.33) | (3.27-24.36) |  |
|  |  | Difficulty falling | 20.0% | 2.2% | 13.04 | 12.87 | <0.001^b^ |
|  |  | asleep or staying | (22/110) | (7/319) | (5.13-33.16) | (4.91-33.72) |  |
|  |  | asleep |  |  |  |  |  |
|  |  | Feeling fearful | 0.0% | 0.9% | 0.00 | 0.00 | 0.996 |
|  |  |  | (0/110) | (3/319) | (0.00-0.00) | (0.00-0.00) |  |
|  |  | Spells of terror or | 25.5% | 0.3% | 108.58 | 134.09 | <0.001^b^ |
|  |  | panic | (28/110) | (1/319) | (14.56-809.90) | (17.41- |  |
|  |  |  |  |  |  | 1032.74) |  |
|  |  | Trembling | 3.6% | 0.9% | 5.98 | 7.91 | 0.028^b^ |
|  |  |  | (4/110) | (3/319) | (1.08-33.12) | (1.25-50.14) |  |
| 267 |  | ^a^ Adjusted for age, gender, education, marital status, number of children, and wealth status. | | | | |  |
| 268 | ^b^ Statistically significant (*p*<0.05). | | |  |  |  |  |
| 269 |  | Nearly all | the physical | and mental health | sequelae were | statistically | significant, |

270 exhibiting a much higher likelihood of occurring in EVD survivors. Some particularly survivor-13

271 specific physical health signs and symptoms were vision [AOR = 24.03 (95% CI, 11.41-50.61),
272 *p* < 0.001], hearing [AOR = 15.82 (95% CI, 4.33-57.82), *p* <0.001], heart problems [AOR =
273 10.70 (95% CI, 4.72-24.26), *p* < 0.001], and numbness of extremities [AOR = 10.62 (95% CI,
274 3.23-34.90), *p* < 0.001]. Mental health signs and symptoms were also significantly higher in
275 EVD survivors versus community members, particularly spells of terror and panic [AOR =
276 134.09 (95% CI, 17.41-1032.74), *p* <0.001] and feeling suddenly scared for no reason [AOR
277 = 64.27 (95% CI, 8.08-511.03), *p* <0.001].

278	Survivors exhibited higher rates of each sequela compared to community members,

279 aside from two with relatively low rates (diarrhea and feeling fearful). As demonstrated in
280 Fig 1, the five most prevalent physical sequalae reported by EVD survivors were joint pain
281 (75.5%), headaches (68.2%), vision problems (44.5%), fatigue/weakness (31.8%), and
282 stomach pain (30.9%). The most prevalent psychological symptoms were spells of terror and
283 panic (25.5%) and difficulty falling asleep or staying asleep (20.0%).
284 **Figure 1. Frequency Distribution of Sequelae in EVD Survivors and Community**
285 **Members.**
286 **Duration of symptoms**

287	For those who reported symptoms present, they were also asked how many years it had

288 been a problem for them. Survivors reported experiencing symptoms for an average of
289 5.18±2.34 years, compared to an average of 2.41±1.80 years for community members. An
290 independent t-test determined that this was statistically significant difference, *p* <0.001.

291	The frequency distribution of responses for EVD survivors and community members

292 also demonstrated skews in the opposite directions (left and right, respectively). EVD survivors
293 are more likely to have experienced symptoms for 6 years (16.5%) or 7 years (48.6%), around
294 the time of the epidemic (see Fig 2).

14

295 **Figure 2. Frequency Distribution of Duration of Symptoms in EVD Survivors and**
296 **Community Members.**
297 **Symptom interference in life**

298	Participants were also asked to rank how much the problem interferes with their lives

299 (on a scale from one to three). An ordinal regression was run to determine the effect of
300 surviving EVD upon ratings of symptom interference in the lives of participants. EVD
301 survivors were found to be 2.91 (95% CI, 2.20-3.83) times more likely to rank their symptoms
302 as causing higher interference than community members after adjusting for age, gender,
303 education, marital status, number of children, and wealth status, a statistically significant effect
304 at *p* < 0.001. The unadjusted OR was 3.05 (95% CI, 2.36-3.94).

305	This is also evident in Fig 3, which illustrates that EVD survivors had a higher

306 frequency of symptoms that they rated as causing interference in their lives. Survivors ranked
307 44.2% of their symptoms as causing a lot of interference, and only listed 7.8% of symptoms as
308 causing no interference at all. Community members, on the other hand, had a relatively normal
309 distribution, with most symptoms (61.6%) causing a little interference.
310 **Figure 3. Frequency Distribution of Symptom Interference in Life in EVD Survivors and**
311 **Community Members.**
312 **Discussion**

313	In this study, we use population-based data from Kenema District, Sierra Leone,

314 collected from EVD survivors and a comparison group, to measure the long-term impact of
315 EVD on physical and mental health. Unlike previous research, this study has a comparison
316 group, takes place more than five years after the outbreak in Eastern Province, and includes a
317 relatively robust sample size of participants.

15

318	The findings strongly indicate that Ebola Virus Disease (EVD) survivors in Kenema

319 District, Sierra Leone still have unmet health needs six years after the epidemic. They are
320 experiencing disproportionately lower health than community members due to significantly
321 higher numbers of post-EVD physical and mental health sequelae. Most of these symptoms
322 have been present since the time of the infection or shortly thereafter, six and seven years prior
323 to survey administration. Of respondents who presented with symptoms, survivors were also
324 significantly more likely to rank their symptoms as causing high interference in their lives. The
325 population-based findings that EVD survivors are still experiencing more physical and mental
326 health sequelae than their community members are extremely compelling, especially given the
327 length of time between the EVD epidemic and data collection.

328	Physical health symptoms reported by EVD survivors, both through the survey and

329 participant observations, aligned with those reported by other studies, although many other
330 studies were restricted to the first few years following the epidemic. Three large-scale cohort
331 studies surveying self-reported physical sequalae of EVD survivors collected data for a range
332 of two to four years after infection, reporting long-term impacts. These studies were the
333 Liberian Ebola Survivor Cohort (2015-19, *n =* 326), PostEboGui in Guinea (2015-16, *n =* 802),
334 and PREVAIL-III in Liberia (2015-17, *n* = 966) [4,5,36,37]. Comparability between studies is
335 limited, as survey tools inquired about different symptoms and only PREVAIL-III included a
336 comparison group, but they generally found some of the same common sequelae of joint pain,
337 headaches, fatigue, and ocular issues.

338	These studies reported a range of prevalences in their populations. Four years after

339 discharge, the Liberian Ebola Survivor Cohort team reported joint pain (39.3%), headaches
340 (32.5%), fatigue (30.8%), and vision problems (18.4%) as the most prominent symptoms in
341 EVD survivors [37]. The PREVAIL-III study reported uveitis (33.3%), headaches (32.6%),
342 joint pain (27.6%), cataracts (13.8%), and muscle pain (12.8%) at the most prevalent symptoms 16
343 in EVD survivors two years after infection, and all were significantly more likely to occur in
344 EVD survivors than close contacts [5]. Two years post-discharge, PostEboGui found general
345 symptoms (25.3%), abdominal pain (17.1%), musculoskeletal (16.8%), and ocular symptoms
346 (6.07%) to be the most common sequelae [4]. While the studies described similar sequelae, all
347 reported lower prevalences compared to our study, which found high rates of joint pain
348 (75.5%), headaches (68.2%), vision problems (44.5%), fatigue/weakness (31.8%), and
349 stomach pain (30.9%). This is an interesting finding, considering that this study occurred six
350 years after the epidemic as opposed to two to four years.

351	Ocular issues are specifically well-documented as a unique sequela in EVD survivors

352 from the West Africa epidemic. Ocular issues were represented in the survey results as a
353 significant concern, as 44.5% of survivors reported vision problems [AOR = 24.03 (95% CI,
354 9.83-47.76), *p*-value <0.001]. Prior literature has described scarring, uveitis leading to eye pain,
355 redness, vision loss, and cataracts in EVD survivors, which often require specialty services at
356 secondary, or even tertiary, facilities [47–49].

357	The few longitudinal studies that have been done have found mixed results in terms of

358 changes over time. The Liberian Ebola Survivor study found that 75.5% of EVD survivors
359 reported one new cardinal symptom since surviving EVD, which 85.8% rated as highly
360 interfering with their lives [37]. The Liberian Ebola Survivor Study also reported that symptom
361 frequency had stabilized and was unchanged at the final follow-up approximately four years
362 after infection. However, PostEboGui and PREVAIL-III noted decreases in sequelae in the
363 years following infection, aside from ocular complications, which increased over time [5,36].
364 As this study does not have a baseline it is impossible to compare, but it is notable that rates
365 are still high, and significantly higher than that of community members, six years post-
366 infection.

17

367	Findings of mental health signs and symptoms varied. Most anxiety- and PTSD-related

368 symptoms listed in the survey were reported by survivors at significantly higher rates than
369 community members, including spells of terror or panic, which was the most prevalent at
370 25.5% and was 129.02 times (95% CI, 16.24-1024.67), *p* < 0.001, more likely to occur in EVD
371 survivors than community members. Marginal rates of EVD survivors and community
372 members reported feeling fearful, and the difference was not significant. In addition, 19.1% of
373 EVD survivors reported feeling depressed or downhearted most or all the time, which was not
374 significantly different from community members.

375	While it is difficult to directly compare these measures to other studies due to

376 differences in scales and diagnostic criteria, these findings align with prior evidence describing
377 long-term mental health impacts of EVD such as anxiety, depression, and PTSD [9]. A study
378 conducted in 2017 with survivors of the 1995 EVD outbreak in Kikwit, Democratic Republic
379 of Congo found significantly higher rates of depression and anxiety in EVD survivors
380 compared to close contacts, 22 years after the epidemic [50]. Depression is the most
381 documented mental illness in EVD survivors, with Lötsch et al. (2017)’s systematic review of
382 long-term neuropsychological sequelae reporting rates of depression ranging from 17 to 50%,
383 with a pooled total of 20% [9]. The authors noted that nearly all the included articles used
384 different methods, some had relatively small samples, and few included comparison groups.
385 Bah et al. (2020) surveyed 197 EVD survivors using the HADs scale in Bombali District, Sierra
386 Leone in 2017 and found rates of 24.9% for anxiety, 47.2% for depression, and 21.8% for
387 PTSD [51]. While this study does not have directly comparable prevalences, it was clear that
388 anxiety and PTSD-related mental health symptoms were far more likely to occur in EVD
389 survivors than community members [AOR = 13.59 (95% CI, 8.15-22.67), *p* < 0.001], aligning
390 with prior research about the neuropsychological impacts of EVD.

18

391	It is worth noting that some of the variations between reported rates may be due to the

392 cultural differences in wording and experiences of mental health, which could be explored
393 through subsequent research. Breakthrough ACTION wrote a formative report based on
394 interviews with EVD survivors in Bombali and Kenema Districts and found that survivors often
395 discussed anxieties or constant worries, describing these feelings as “worried,” “stressed,”
396 “embittered,” “sad,” “angry,” “disturbed,” and “frustrated” [52]. Utilizing surveys previously
397 validated and adapted to similar populations and cultural contexts may provide more accurate
398 insight into the mental health concerns of EVD survivors.

399	Although it cannot be ascertained through findings from the cross-sectional survey, it

400 is also worth noting the potential socioeconomic impacts of long-term physical and mental
401 health sequelae in EVD survivors. While prior studies exploring the connection between EVD-
402 related health status and the livelihoods of survivors are limited, Karafillakis et al. (2015)
403 interviewed and surveyed 28 EVD survivors in Sierra Leone directly after the outbreak, finding
404 that most interviewees had lost their jobs, with some attributing it to a lack of strength [18]. In
405 addition, the disability of EVD survivors has been documented. Bai James et al. (2020) found
406 that EVD survivors in Sierra Leone had the lowest SF-36 scores in the physical role limitation
407 domains due to physical and emotional health [16]. Jagadesh et al. (2018) found that EVD
408 survivors in Freetown had significantly more physical disability than their close contacts,
409 including limitations in walking 500 m (74.1%) and climbing 12 steps (85.2%), both
410 significantly higher than controls at *p* < 0,001 [49].

411	These findings may also demonstrate the bidirectional influences between physical and

412 mental health that have been observed in numerous other illnesses [53]. Physical illness can
413 lead to or worsen mental illness and mental illness can lead to or worsen physical illness,
414 reflecting a holistic experience of overall health [53].

19

415	Mental illness can be influenced directly by EVD-related experiences and grief, and

416 indirectly by experiences with physical sequelae post-EVD. The comorbidity of chronic
417 physical and mental illnesses, primarily major depressive disorder, has been described
418 extensively in literature [53]. This includes patients with non-communicable diseases such as
419 heart disease, cancer, diabetes, lung disease, and chronic pain (106–112), and patients with
420 communicable diseases such as HIV [61,62].

421	In addition, it is documented that mental health can impact physical health. For

422 example, anxiety and depression have been tied to an increased prevalence of lower back pain
423 [63], cardiovascular disease [57], and cancer [54,59]. It can also worsen existing physical
424 conditions and result in poorer prognoses [59]. Breakthrough ACTION’s report on the status
425 of EVD survivors in Kenema and Bombali Districts (2021) also reported psychosomatic
426 symptoms in EVD survivors, for example describing headaches, blindness, sleeplessness,
427 dreams, and feelings of sadness that specifically arose on anniversaries of days where they
428 contracted the virus or when friends and family members passed away [52].

429	Physical and mental health are influenced by numerous external factors, as well as by

430 each other. When looking at sequelae experienced by EVD survivors, it is important to
431 acknowledge that they are not standalone experiences and likely interact with each other,
432 resulting in survivors who have multiple comorbidities and are experiencing poor health
433 overall. Thus, it seems logical to have a holistic perspective towards health, understanding that
434 physical, mental, and social health are all contributing to the overall health and wellbeing of
435 EVD survivors. It is also important to acknowledge that research about interactions between
436 physical and mental health is limited in Sierra Leone and West Africa, and experiences likely
437 vary across cultures.

438	There are numerous strengths of the study, primarily being that it was population-based

439 and had a comparison group sampled from the communities of EVD survivors. The response 20
440 rate was high at 76.9% of EVD survivors over 15 years of age in Kenema District, and those
441 who were not surveyed were not notably different in age and gender compared to those who
442 were. The study is likely to be generalizable to the experiences of EVD survivors in Kenema
443 District and similar areas impacted by the West Africa outbreak, with some applicability to
444 other EVD outbreaks in similar settings and similar epidemics in West Africa.
445 **Limitations and bias**

446	Selection bias may have occurred to some extent. While recruitment by the SLAES

447 executive board allowed the team to survey the majority of EVD survivors in Kenema District,
448 those who were not included due to being out of town or unreachable may have offered
449 different perspectives. In addition, the EVD survivors surveyed were laboratory-verified cases
450 and had certificates, whereas those who did not get tested and go to an ETU would not be
451 included in the survey.

452	We also note that responses were all self-reported and not verified by a physician. While

453 there are strengths to capturing the perceptions of survivors concerning their health status, these
454 perceptions are subject to bias. On one hand, EVD survivors may have responded cautiously
455 to potentially sensitive and stigmatizing topics such as mental health and sexual dysfunction
456 due to desirability bias, whereas they may have been inclined to over-report health concerns
457 due to the dynamics of humanitarian aid and the history of individuals receiving funding based
458 on sharing their health status [64].

459	As the study design was cross-sectional, causality cannot be established. This is an

460 observational, descriptive study of the perceptions of EVD survivors about experiences with
461 post-EVD sequelae, overall health and wellbeing, and health care access compared to their
462 community members. The study had strengths in establishing associations between
463 survivorship and health outcomes with a high statistical power due to its robust, population-
464 based sample and comparison group. However, a longitudinal cohort study would be ideal to 21
465 establish causality in an observational study and should be considered in future post-epidemic
466 research.
467 **Conclusions**

468	Findings from this study indicate that EVD survivors require a comprehensive, long-

469 term package of care built into existing health care systems with a strong consideration of
470 sustainability. Many EVD survivor programmes ceased to exist after international attention,
471 and thus funding declined, leaving survivors suddenly unable to access prior sources of care.
472 Given the increasing threat of emerging infectious diseases, including recent outbreaks of
473 COVID-19, EVD in DRC and Uganda, and Marburg in Equatorial Guinea, it is highly relevant
474 to consider the long-term impacts of diseases upon the health status and social determinants of
475 health (including socioeconomic status) of survivors.

476	Given the finding that EVD survivors are significantly more likely to experience more

477 physical and mental health sequelae of increased severity six years after the epidemic, it is clear
478 that epidemic responses need to integrate a longer-term, health systems strengthening and
479 health equity-based approach to providing care for survivors. In addition, further research
480 should be undertaken to determine health care-seeking behaviors of EVD survivors and
481 availability of health care services to identify potential gaps and areas that could be
482 strengthened through international and national programming and guidelines.

22

483 **References**
484 1. Centers for Disease Control and Prevention. 2014-2016 Ebola Outbreak in West Africa.
485 In: Centers for Disease Control and Prevention [Internet]. 3 Aug 2019 [cited 24 Jan
486 2021]. Available: https://www.cdc.gov/vhf/ebola/history/2014-2016-
487 outbreak/index.html
488 2. Richards P. Ebola: How a People’s Science Helped End an Epidemic. London, UK: Zell
489 Books; 2016.
490 3. Bond NG, Grant DS, Himmelfarb ST, Engel EJ, Al-Hasan F, Gbakie M, et al. Post-Ebola
491 Syndrome Presents With Multiple Overlapping Symptom Clusters: Evidence From an
492 Ongoing Cohort Study in Eastern Sierra Leone. Clinical Infectious Diseases. 2021;73:
493 1046–1054. doi:10.1093/cid/ciab267
494 4. Etard J-F, Sow MS, Leroy S, Touré A, Taverne B, Keita AK, et al. Multidisciplinary
495 assessment of post-Ebola sequelae in Guinea (Postebogui): an observational cohort
496 study. The Lancet Infectious Diseases. 2017;17: 545–552. doi:10.1016/S1473-
497 3099(16)30516-3
498 5. The PREVAIL III Study Group. A Longitudinal Study of Ebola Sequelae in Liberia. N
499 Engl J Med. 2019;380: 924–934. doi:10.1056/NEJMoa1805435
500 6. Godwin CL, Wohl DA, Fischer 2nd WA, Singh K, Hawks DA, Devore EE, et al.
501 Reproductive health sequelae among women who survived Ebola virus disease in
502 Liberia. Int J Gynecol Obstet. 2019;146: 212–217. doi:10.1002/ijgo.12858
503 7. Keita MM, Taverne B, Sy Savané S, March L, Doukoure M, Sow MS, et al. Depressive
504 symptoms among survivors of Ebola virus disease in Conakry (Guinea): preliminary
505 results of the PostEboGui cohort. BMC Psychiatry. 2017;17: 127. doi:10.1186/s12888-
506 017-1280-8

23

507 8. Guetiya Wadoum RE, Samin A, Mafopa NG, Giovanetti M, Russo G, Turay P, et al.
508 Mobile health clinic for the medical management of clinical sequelae experienced by
509 survivors of the 2013–2016 Ebola virus disease outbreak in Sierra Leone, West Africa.
510 European Journal of Clinical Microbiology & Infectious Diseases. 2017;36: 2193–2200.
511 9. Lötsch F, Schnyder J, Goorhuis A, Grobusch MP. Neuropsychological long-term
512 sequelae of Ebola virus disease survivors – A systematic review. Travel Medicine and
513 Infectious Disease. 2017;18: 18–23. doi:10.1016/j.tmaid.2017.05.001
514 10. Nanyonga M, Saidu J, Ramsay A, Shindo N, Bausch DG. Sequelae of Ebola Virus
515 Disease, Kenema District, Sierra Leone. Clin Infect Dis. 2016;62: 125–126.
516 doi:10.1093/cid/civ795
517 11. Qureshi AI, Chughtai M, Loua TO, Pe Kolie J, Camara HFS, Ishfaq MF, et al. Study of
518 Ebola Virus Disease Survivors in Guinea. Clin Infect Dis. 2015;61: 1035–1042.
519 doi:10.1093/cid/civ453
520 12. Hugo M, Declerck H, Fitzpatrick G, Severy N, Gbabai OB-M, Decroo T, et al. Post-
521 Traumatic Stress Reactions in Ebola Virus Disease Survivors in Sierra Leone. Emerg
522 Med (Los Angel). 2015;05. doi:10.4172/2165-7548.1000285
523 13. Jalloh MF, Li W, Bunnell RE, Ethier KA, O’Leary A, Hageman KM, et al. Impact of
524 Ebola experiences and risk perceptions on mental health in Sierra Leone, July 2015.
525 BMJ Glob Health. 2018;3: e000471. doi:10.1136/bmjgh-2017-000471
526 14. Kuriansky J. The Psychosocial Aspects of a Deadly Epidemic: What Ebola Has Taught
527 Us about Holistic Healing (Practical and Applied Psychology). 1st edition. Santa
528 Barbara, CA; Denver, CO: Praeger; 2016.
529 15. Overholt L, Wohl DA, Fischer WA, Westreich D, Tozay S, Reeves E, et al. Stigma and
530 Ebola survivorship in Liberia: Results from a longitudinal cohort study. PLoS ONE.
531 2018;13. doi:10.1371/journal.pone.0206595

24

532 16. James PB, Wardle J, Steel A, Adams J. An assessment of Ebola-related stigma and its
533 association with informal healthcare utilisation among Ebola survivors in Sierra Leone:
534 a cross-sectional study. BMC Public Health. 2020;20: 182. doi:10.1186/s12889-020-
535 8279-7
536 17. Richards P, Amara J, Ferme MC, Kamara P, Mokuwa E, Sheriff AI, et al. Social
537 Pathways for Ebola Virus Disease in Rural Sierra Leone, and Some Implications for
538 Containment. Powers AM, editor. PLoS Negl Trop Dis. 2015;9: e0003567.
539 doi:10.1371/journal.pntd.0003567
540 18. Karafillakis E, Jalloh MF, Nuriddin A, Larson HJ, Whitworth J, Lees S, et al. ‘Once there
541 is life, there is hope’ Ebola survivors’ experiences, behaviours and attitudes in Sierra
542 Leone, 2015. BMJ Glob Health. 2016;1: e000108. doi:10.1136/bmjgh-2016-000108
543 19. Matua GA, Wal DMV der. Living Under the Constant Threat of Ebola: A
544 Phenomenological Study of Survivors and Family Caregivers During an Ebola
545 Outbreak. Journal of Nursing Research. 2015;23: 217–224.
546 doi:10.1097/jnr.0000000000000116
547 20. Rabelo I, Lee V, Fallah MP, Massaquoi M, Evlampidou I, Crestani R, et al.
548 Psychological Distress among Ebola Survivors Discharged from an Ebola Treatment
549 Unit in Monrovia, Liberia – A Qualitative Study. Front Public Health. 2016;4.
550 doi:10.3389/fpubh.2016.00142
551 21. Schwerdtle PM, De Clerck V, Plummer V. Experiences of Ebola Survivors: Causes of
552 Distress and Sources of Resilience. Prehosp Disaster med. 2017;32: 234–239.
553 doi:10.1017/S1049023X17000073
554 22. Groupe d’étude PostEboGui, Sow S, Desclaux A, Taverne B. Ebola en Guinée : formes
555 de la stigmatisation des acteurs de santé survivants. Bull Soc Pathol Exot. 2016;109:
556 309–313. doi:10.1007/s13149-016-0510-5

25

557 23. Yamanis T, Nolan E, Shepler S. Fears and Misperceptions of the Ebola Response System
558 during the 2014-2015 Outbreak in Sierra Leone. Kasper M, editor. PLoS Negl Trop Dis.
559 2016;10: e0005077. doi:10.1371/journal.pntd.0005077
560 24. Bowen L, Smith B, Steinbach S, Billioux B, Summers A, Azodi S, et al. Survivors of
561 Ebola Virus Disease Have Persistent Neurological Deficits (S53.003). Neurology.
562 2016;86: S53.003. doi:10.1212/WNL.86.16_supplement.S53.003
563 25. Deen GF, Broutet N, Xu W, Knust B, Sesay FR, McDonald SLR, et al. Ebola RNA
564 Persistence in Semen of Ebola Virus Disease Survivors — Final Report. N Engl J Med.
565 2017;377: 1428–1437. doi:10.1056/NEJMoa1511410
566 26. Hewlett BS, Amola RP. Cultural Contexts of Ebola in Northern Uganda. Emerg Infect
567 Dis. 2003;9: 1242–1248. doi:10.3201/eid0910.020493
568 27. Mohammed A, Sheikh TL, Gidado S, Poggensee G, Nguku P, Olayinka A, et al. An
569 evaluation of psychological distress and social support of survivors and contacts of
570 Ebola virus disease infection and their relatives in Lagos, Nigeria: a cross sectional
571 study − 2014. BMC Public Health. 2015;15: 824. doi:10.1186/s12889-015-2167-6
572 28. De Roo A, Ado B, Rose B, Guimard Y, Fonck K, Colebunders R. Survey among
573 survivors of the 1995 Ebola epidemic in Kikwit, Democratic Republic of Congo: their
574 feelings and experiences. Tropical Med Int Health. 1998;3: 883–885.
575 doi:10.1046/j.1365-3156.1998.00322.x
576 29. Sack K, Fink S, Belluck P, Nossiter A. How Ebola Roared Back. New York Times. 29
577 Dec 2014: Page 1.
578 30. Centers for Disease Control and Prevention. 2014-2016 Ebola Outbreak in West Africa,
579 Case Counts. 2016. Available: https://www.cdc.gov/vhf/ebola/history/2014-2016-
580 outbreak/case-counts.html

26

581 31. Richards P, Mokuwa GA, Vandi A, Mayhew SH, Ebola Gbalo Research Team. Re-
582 analysing Ebola spread in Sierra Leone: The importance of local social dynamics.
583 Hodges MH, editor. PLoS ONE. 2020;15: e0234823. doi:10.1371/journal.pone.0234823
584 32. Turner-Bowker D, Hogue SJ. Short Form 12 Health Survey (SF-12). In: Michalos AC,
585 editor. Encyclopedia of Quality of Life and Well-Being Research. Dordrecht: Springer
586 Netherlands; 2014. pp. 5954–5957. doi:10.1007/978-94-007-0753-5_2698
587 33. Hjelm L, Mathiassen A, Miller D, Wadhwa A. VAM Guidance Paper: Creation of a
588 Wealth Index. World Food Programme; 2017. Available:
589 https://docs.wfp.org/api/documents/WFP-0000022418/download/
590 34. The DHS Program. Wealth Index Construction. In: The DHS Program [Internet]. [cited
591 26 Aug 2022]. Available: https://dhsprogram.com/topics/wealth-index/Wealth-Index-
592 Construction.cfm
593 35. Jacob ST, Crozier I, Fischer WA, Hewlett A, Kraft CS, Vega M-A de L, et al. Ebola virus
594 disease. Nat Rev Dis Primers. 2020;6: 13. doi:10.1038/s41572-020-0147-3
595 36. Diallo MSK, Toure A, Sow MS, Kpamou C, Keita AK, Taverne B, et al. Understanding
596 Long-term Evolution and Predictors of Sequelae of Ebola Virus Disease Survivors in
597 Guinea: A 48-Month Prospective, Longitudinal Cohort Study (PostEboGui). Clinical
598 Infectious Diseases. 2021;73: 2166–2174. doi:10.1093/cid/ciab168
599 37. Tozay S, Fischer WA, Wohl DA, Kilpatrick K, Zou F, Reeves E, et al. Long-term
600 Complications of Ebola Virus Disease: Prevalence and Predictors of Major Symptoms
601 and the Role of Inflammation. Clinical Infectious Diseases. 2020;71: 1749–1755.
602 doi:10.1093/cid/ciz1062
603 38. Thompson AE, Anisimowicz Y, Miedema B, Hogg W, Wodchis WP, Aubrey-Bassler K.
604 The influence of gender and other patient characteristics on health care-seeking

27

605 behaviour: a QUALICOPC study. BMC Fam Pract. 2016;17: 38. doi:10.1186/s12875-
606 016-0440-0
607 39. Atchessi N, Ridde V, Abimbola S, Zunzunegui M-V. Factors associated with the
608 healthcare-seeking behaviour of older people in Nigeria. Archives of Gerontology and
609 Geriatrics. 2018;79: 1–7. doi:10.1016/j.archger.2018.07.015
610 40. Grépin KA, Poirier MJP, Fox AM. The socio-economic distribution of exposure to Ebola:
611 Survey evidence from Liberia and Sierra Leone. SSM - Population Health. 2020;10:
612 100472. doi:10.1016/j.ssmph.2019.100472
613 41. Liwin LK, Houle B. The effects of household and community context on mortality
614 among children under five in Sierra Leone: Evidence from the 2013 Demographic and
615 Health Survey. DemRes. 2019;40: 279–306. doi:10.4054/DemRes.2019.40.11
616 42. Bognini JD, Samadoulougou S, Ouedraogo M, Kangoye TD, Van Malderen C, Tinto H,
617 et al. Socioeconomic inequalities in curative healthcare-seeking for children under five
618 before and after the free healthcare initiative in Sierra Leone: analysis of population-
619 based survey data. Int J Equity Health. 2021;20: 124. doi:10.1186/s12939-021-01474-7
620 43. Squire JS, Hann K, Denisiuk O, Kamara M, Tamang D, Zachariah R. The Ebola outbreak
621 and staffing in public health facilities in rural Sierra Leone: who is left to do the job?
622 Public Health Action. 2017; 8.
623 44. World Medical Association. WMA Declaration of Helsinki - Ethical Principles for
624 Medical Research Involving Human Subjects. 1964. Available:
625 https://www.wma.net/policies-post/wma-declaration-of-helsinki-ethical-principles-for-
626 medical-research-involving-human-subjects/
627 45. Regulation (EU) 2016/679 of the European Parliament and of the Council of 27 April
628 2016 on the protection of natural persons with regard to the processing of personal data

28

629 and on the free movement of such data, and repealing Directive 95/46/EC. OJ 2016 L
630 119/1 2016. Available: https://gdpr-info.eu
631 46. Bonuedi I, Kornher L, Gerber N. Agricultural seasonality, market access, and food
632 security in Sierra Leone. Food Sec. 2022;14: 471–494. doi:10.1007/s12571-021-01242-
633 z
634 47. Shantha JG, Hayek BR, Crozier I, Gargu C, Dolo R, Brown J, et al. Development of a
635 screening eye clinic for Ebola virus disease survivors: Lessons learned and rapid
636 implementation at ELWA Hospital in Monrovia, Liberia 2015. Schibler M, editor. PLoS
637 Negl Trop Dis. 2019;13: e0007209. doi:10.1371/journal.pntd.0007209
638 48. Shantha JG, Crozier I, Hayek BR, Bruce BB, Gargu C, Brown J, et al. Ophthalmic
639 Manifestations and Causes of Vision Impairment in Ebola Virus Disease Survivors in
640 Monrovia, Liberia. Ophthalmology. 2017;124: 170–177.
641 doi:10.1016/j.ophtha.2016.10.011
642 49. Jagadesh S, Sevalie S, Fatoma R, Sesay F, Sahr F, Faragher B, et al. Disability Among
643 Ebola Survivors and Their Close Contacts in Sierra Leone: A Retrospective Case-
644 Controlled Cohort Study. Clinical Infectious Diseases. 2018;66: 131–133.
645 doi:10.1093/cid/cix705
646 50. Kelly JD, Hoff NA, Spencer D, Musene K, Bramble MS, McIlwain D, et al.
647 Neurological, Cognitive, and Psychological Findings Among Survivors of Ebola Virus
648 Disease From the 1995 Ebola Outbreak in Kikwit, Democratic Republic of Congo: A
649 Cross-sectional Study. Clinical Infectious Diseases. 2019;68: 1388–1393.
650 doi:10.1093/cid/ciy677
651 51. Bah AJ, James PB, Bah N, Sesay AB, Sevalie S, Kanu JS. Prevalence of anxiety,
652 depression and post-traumatic stress disorder among Ebola survivors in northern Sierra

29

653 Leone: a cross-sectional study. BMC Public Health. 2020;20: 1391.
654 doi:10.1186/s12889-020-09507-6
655 52. Kumoji K, Bride M, Dickenson T, Clayton S, Nyuma G, Bockarie F, et al. Adaptation,
656 Experiences, and Support Needs of Survivors of Ebola Virus Disease in Bombali and
657 Kenema Districts of Sierra Leone: Formative Assessment in Kenema and Bombali
658 Districts. Breakthrough ACTION Sierra Leone Program; 2021.
659 53. Parrish E. Comorbidity of mental illness and chronic physical illness: A diagnostic and
660 treatment conundrum. Perspect Psychiatr Care. 2018;54: 339–340.
661 doi:10.1111/ppc.12311
662 54. Rathi M, Singh S, Yadav S, Budania S. Depression and cancer: An update. J Sci Soc.
663 2014;41: 232. doi:10.4103/0974-5009.141258
664 55. Habtewold TD, Islam MdA, Radie YT, Tegegne BS. Comorbidity of depression and
665 diabetes: an application of biopsychosocial model. Int J Ment Health Syst. 2016;10: 74.
666 doi:10.1186/s13033-016-0106-2
667 56. Ohrnberger J, Fichera E, Sutton M. The dynamics of physical and mental health in the
668 older population. The Journal of the Economics of Ageing. 2017;9: 52–62.
669 doi:10.1016/j.jeoa.2016.07.002
670 57. Penninx BWJH. Depression and cardiovascular disease: Epidemiological evidence on
671 their linking mechanisms. Neuroscience & Biobehavioral Reviews. 2017;74: 277–286.
672 doi:10.1016/j.neubiorev.2016.07.003
673 58. Schmitt A, Reimer A, Kulzer B, Haak T, Gahr A, Hermanns N. Negative association
674 between depression and diabetes control only when accompanied by diabetes-specific
675 distress. J Behav Med. 2015;38: 556–564. doi:10.1007/s10865-014-9604-3

30

676 59. Sotelo JL, Musselman D, Nemeroff C. The biology of depression in cancer and the
677 relationship between depression and cancer progression. International Review of
678 Psychiatry. 2014;26: 16–30. doi:10.3109/09540261.2013.875891
679 60. Woo AK. Depression and Anxiety in Pain. Reviews in Pain. 2010;4: 8–12.
680 doi:10.1177/204946371000400103
681 61. LeMasters K, Dussault J, Barrington C, Bengtson A, Gaynes B, Go V, et al. “Pain in my
682 heart”: Understanding perinatal depression among women living with HIV in Malawi.
683 Montazeri A, editor. PLoS ONE. 2020;15: e0227935.
684 doi:10.1371/journal.pone.0227935
685 62. Singer M, Clair S. Syndemics and Public Health: Reconceptualizing Disease in Bio-
686 Social Context. Medical Anthropology Quarterly. 2003;17: 423–441.
687 doi:10.1525/maq.2003.17.4.423
688 63. Pinheiro MB, Ferreira ML, Refshauge K, Colodro-Conde L, Carrillo E, Hopper JL, et al.
689 Genetics and the environment affect the relationship between depression and low back
690 pain: a co-twin control study of Spanish twins. Pain. 2015;156: 496–503.
691 doi:10.1097/01.j.pain.0000460330.56256.25
692 64. Nguyen V-K. The Republic of Therapy. Durham and London: Duke University Press;
693 2010.

694

31

Fig1	[Click here to access/download;Figure;Fig1.tif](https://www.editorialmanager.com/pgph/download.aspx?id=295028&guid=8a3e3d74-40a3-4fe7-8aec-4867040fe493&scheme=1)

Fig2	[Click here to access/download;Figure;Fig2.tif](https://www.editorialmanager.com/pgph/download.aspx?id=295029&guid=c89ba15f-e565-4d73-9168-e0a2b4f9a064&scheme=1)

Fig3	[Click here to access/download;Figure;Fig3.tif](https://www.editorialmanager.com/pgph/download.aspx?id=295030&guid=19fa7015-d538-4699-89d2-7ee7825c6199&scheme=1)
