# Supplementary material for: Long-term physical and mental health outcomes of Ebola Virus Disease survivors in Kenema District, Sierra Leone: A cross-sectional survey
Source: PLOS Glob Public Health. 2024 Nov 4;4(11):e0003421. doi: 10.1371/journal.pgph.0003421 (PMC11534246; doi:10.1371/journal.pgph.0003421)
Supplement: Attachment — Submitted filename: Responses to reviewers comments.docx [file pgph.0003421.s005.docx]

**Ref.: PGPH-D-24-00262**

**Long-term physical and mental health outcomes of Ebola Virus Disease survivors in Kenema District, Sierra Leone: a cross-sectional survey**

We thank the editor and reviewers for their thoughtful comments and feedback. We have listed the editor’s and both reviewers’ comments in bold with our responses in plain font underneath each individual comment.

**Editor Comments :**

1. **Please provide a clear study justification given that a lot of studies have been conducted on this topic. what new knowledge is the study providing**

We have now expanded the study justification in the text. Prior studies about the long-term health impacts of EVD survivors have either not included a comparison group or have used a control group of close contacts, who may also be subject to negative physical and mental health outcomes due to the household-based nature of the spread of EVD (**e.g., Bond et al. 2021**). In this study, we compared the health of EVD survivors with a population-based sample of community members- a strength that is pointed out by reviewer #2. Using this approach, we found significantly higher rates of symptoms present for a longer period of time and with higher severity in EVD survivors compared to community members, six years after the epidemic. This demonstrates an urgent and pressing need to consider the long-term impacts of Ebola and infectious disease epidemics in general when planning responses.

Reference: Bond, N. G., Grant, D. S., Himmelfarb, S. T., Engel, E. J., Al-Hasan, F., Gbakie, M., ... & Schieffelin, J. S. (2021). Post-Ebola syndrome presents with multiple overlapping symptom clusters: evidence from an ongoing cohort study in Eastern Sierra Leone. Clinical Infectious Diseases, 73(6), 1046-1054.

2. **The reviewers were concerned about how the symptoms were measured and the how the data was statistically analysed. I suggest you pay close attention to that as this can be serious ground for rejection.**

We appreciate the comments from both reviewers about measurement, sampling, and analysis, as they have helped to improve this research. In response, we have made several updates to this manuscript.

First, we have updated the analytic method used for the symptom count data from Poisson to negative binomial regression, as suggested by both reviewers (and the results are substantively identical to previous).

Second, we agree with reviewer #2 that an important asset of this work is the population-based control group, since no previous research has used this approach. We have expanded on our sampling methods, and the description of how this approach contributes to the literature.

Third, we have addressed other concerns about measurement, goodness of fit, missing data, and other comments on the analysis from both reviewers.

3. **Another issue that need to be addressed is why the research team decided to use community members as comparison group. why was close family members of EVD survivors were considered**

Our goal in this research was to identify the long-term impact of EVD infection on physical and health outcomes. To do so, we needed a study design that (1) measured longer-term health outcomes among EVD survivors, and (2) included a population-based control group to compare with the EVD survivors. This population-based control group serves the purpose of accounting for other factors that may impact health for residents of Kenema district over the long term, so any differences between groups are due to EVD infection and not other health-related factors. In other words, there may be other factors that influence physical and mental health of Kenema district residents over time, besides EVD, so we compare the EVD survivors with a population-based non EVD group, so that any differences with the EVD group should be due primarily to EVD instead of other health trends and influences.

Similarly, we feel that close family members of EVD survivors are not an appropriate control group, since they share many characteristics of the survivors- for example, since EVD primarily spread through close contacts, they have a likelihood of having similar experiences of, e.g., grief, loss, reduction in wealth status, and, therefore, potential of exhibiting physical and mental health symptoms. Instead, population-based community members were used as a comparison group for the reasons stated above.

**Reviewer #1 (we note that responses to most of Reviewer #1’s comments are included directly in the tracked version of the manuscript)**

**There is a need to provide more insights into the finding.**

We have expanded the discussion section to enhance the contribution of this study.

**The presence of over dispersion in the poisson regression model is a concern and is due to the limited data that was used in this study. However this can be handled by a quasi poisson regression.**

We appreciate this comment and have updated our methods to use a negative binomial regression model to negate the impacts of overdispersion and to increase the goodness of fit. The substantive interpretation of our results remains the same after this update.

**Also the demographic statistics are too basic. so much were mentioned in the methodology section about the Test statistics used in this study but less is Test statistics results were presented.**
The demographic statistics described in this study are the standard set that are typically used in this and related research- age, gender, marital status, wealth, number of children, urban/rural residence, etc. We are open to suggestions for exploring other demographic characteristics in this research. Regarding the test statistics, we included p-values and 95% confidence intervals for all statistical tests between populations but are also open to suggestions for expanding our presentation of results.

**Line 27: Cross sectional that made use of EVD survivor population data??? Compared 27 vs**

Our study used a registry of EVD survivors to identify this group for survey interviews. Data from this registry was not used for the analysis in this research. The methodology (including sampling methods) has now been revised to make this clearer.

**Line 28: Non EVD survivor community members?**

This has been edited to clarify that community members lived in EVD-affected areas but were never diagnosed with EVD. Further details are in the sampling section.

**Line 32: Insert all p values by the corresponding 95%CI**

We have added p-values next to the 95% confidence intervals.

**Line 36: Interesting findings. I was expecting to read reproduction issues as reported health symptoms**

We agree that it is important to examine reproductive health outcomes as well, since these will very likely differ between EVD survivors and the general population. This is the topic of a future and separate analysis, in order to devote the appropriate attention to this topic.

**Line 93: While this historic narration is absolutely correct yet the period of EVD outbreak needs reviewing. The WA Ebola outbreak actually started in December 2013. The infant patient boy Emil died on 6th December 2013 in Gueckedou, Guinea**

We appreciate this comment. The time period of the outbreak has been changed to 2013-2016 throughout the manuscript.

**Line 118: (n = ????)**

Details about the number of EVD survivors in the database have now been added to the manuscript.

**Line 119: Where does 143 comes from? It is good to make it clear to the readers how man**

The composition of the EVD survivor registry has now been detailed in lines 120-123.

**Line 120: Were the control group participants living in the same communities of the EVD survivors prior to/during EVD outbreak period?**

The control group participants were indeed living in the same communities of the EVD survivors prior to/during the outbreak period.

**Line 169: Poisson regression model is not specifically used to model skewed dependent variables**

We agree. This has now been corrected to clarify that the Poisson model (now changed to a negative binomial regression) was selected due to the nature of the dependent variable as count data.

**Line 173: kindly Check font sizes throughout manuscript**

We have ensured that font sizes are consistent throughout the manuscript (Times New Roman size 12, Times New Roman size 10 in tables).

**Line 174: with the presence of over-dispersion of any degree the best way out to improve your model is to use a quasi poisson model**

We appreciate this suggestion and have updated to include a negative binomial regression model to address the possibility of over-dispersion.

**Line 174: the best statistics to test for over-dispersion in passion model is use Wald or score test, or the likelihood ratio test.**

As mentioned above, we agree and have updated our models.

**Line 191: would??**

We have deleted this statement since it is not relevant to the analysis.

**Line 210: This looks like methods/Analytical methods and not Result**

Noted. This has been moved to the “Analytic methods” section.

**Table 1: Kindly format the entire manuscript**

We have updated the formatting of the manuscript to ensure that font type and size and spacing is all consistent.

**Table 1: Interesting result especially so when education level and current wealth status are measuring the same thing**

We examined the relationship between education and wealth, found that these measures are correlated to some extent but not multicollinear, which suggests that they are capturing different concepts.

**Line 226: It helps if author can state the appropriate measures of central tendency and dispersion are stated**

We have provided more detail on the distributions of these measures in Table 1.

**Line 229: This can move over to limitation /discussion section**

Noted; this has been removed from this section.

**Line 250: Prediction or association???**

This has been changed to “association”.

**Line 278: Replace rate with an appropriate term.**

We have changed “rate” to “levels”.

**Line 387: Prevalence rate?**

This has been changed to “levels” throughout this section.

**Line 451: Any reasons/s?**

We have elaborated on this limitation.

**Reviewer #2**

**Abstract**

**2013-2016 epidemic**

We have changed to 2013-2016 epidemic in all cases.

**Nb cases: more than 2 8000 (cf introduction, ref 1 ; [1] WHO Ebola Response Team, After Ebola in West Africa — Unpredictable Risks, Preventable Epidemics, N. Engl. J. Med. 375 (2016) 587–596.)**

We agree and have revised to match the figures in the introduction (28,000 cases in total, rather than the 14,000 cases in Sierra Leone).

**Limited prior research : replace by a few studies**

Noted; this has been replaced.

**Introduction**

**Ebola is no longer clasified as an hemmorrhagic fever by WHO: prefer EVD;**

We have changed the wording to “severe illness.”

**"2014-2016 EVD epidemic": change to "2013-2016 EVD epidemic";**

We have changed the reference to “2013-2016 EVD epidemic” in all cases.

**14124 cases = 8706 confirmed + 287 probable + 5131 suspected**

This has now been specified in the paper.

**CFR: nb of deaths in SL = 3956 but 3589 among 8706 confirmed --> CFR among confirmed cases = 41% --> to be precised**

This has also now been specified.

**"The vast majority of research on the impacts of Ebola infection is qualitative and used convenience samples". True for some studies looking at psychological consequences but not true given large survivors cohorts, for example:**

**Guinea/Postebogui cohort (n=802): Etard in ref 4 ; Diallo in ref 36.**

**Sierra Leone/Kerry Town ETC (n=151): K. Wing, S. Oza, C. Houlihan, J.R. Glynn, S. Irvine, C.E. Warrell, A.J.H. Simpson, S. Boufkhed, A. Sesay, L. Vandi, S.C. Sebba, P. Shetty, R. Cummings, F. Checchi, C.R. McGowan, Surviving Ebola: A historical cohort study of Ebola mortality and survival in Sierra Leone 2014-2015, PLoS One. 13 (2018) 1–17. https://doi.org/10.1371/journal.pone.0209655;**

**H. Bower, E. Smout, M.S. Bangura, O. Kamara, C. Turay, S. Johnson, S. Oza, F. Checchi, J.R. Glynn, Deaths, late deaths, and role of infecting dose in Ebola virus disease in Sierra Leone: retrospective cohort study, BMJ. (2016).** [**https://doi.org/10.1136/bmj.i2403**](https://doi.org/10.1136/bmj.i2403)**.**

**Liberia/PREVAIL III (n=966) in ref 5 ; Longitudinal Liberian Ebola Survivor Study (n=299) in L. Overholt, D.A. Wohl, W.A. Fischer, D. Westreich, S. Tozay, E. Reeves, K. Pewu, D. Adjasso, D. Hoover, C. Merenbloom, H. Johnson, G. Williams, T. Conneh, J. Diggs, A. Buller, D. McMillian, D. Hawks, K. Dube, J. Brown, Stigma and Ebola survivorship in Liberia: Results from a longitudinal cohort study, PLoS One. 13 (2018) 1–13.** [**https://doi.org/10.1371/journal.pone.0206595**](https://doi.org/10.1371/journal.pone.0206595)**.**

We appreciate this comment and agree that the body of research on the impact of EVD on physical and mental health extends beyond the qualitative work. We have updated the manuscript to describe the quantitative studies listed above, have removed the statement that “the vast majority of research” is qualitative, and better described how our research fits within these other studies.

**"In addition, much of the quantitative research on the social and demographic impacts of Ebola was conducted with small samples of less than 120 participants...": ref ?**

We have removed this statement as part of our overall effort to better describe the contribution of this study.

**"research has only examined the impacts of Ebola infection occurring shortly after recovery.": wrong, cf: prospective Postebogui cohort S.M.K. Diallo, A. Toure, M.S. Sow, C. Kpamou, A.K. Keita, B. Taverne, M. Peeters, P. Msellati, T.A. Barry, J.F. Etard, R. Ecochard, E. Delaporte. Understanding Long-term Evolution and Predictors of Sequelae of Ebola Virus Disease Survivors in Guinea: A 48-month prospective, longitudinal cohort study (PosteboguI), Clin. Infect. Dis. 73 (2021) 2166–2174.** [**https://doi.org/10.1093/cid/ciab168**](https://doi.org/10.1093/cid/ciab168)**.** -

We appreciate this correction and agree. We have removed this statement and provided more detail on how some studies such as the one above have followed EVD survivors over time.

**Methods**

**"The 2014-2016 West Africa epidemic...": change to 2013-**

Changed to 2013-2016 in all cases.

**- "the virus quickly spread to Sierra Leone and Liberia [29].": EVD spread in Kailahun district in eastern Sierra Leone in May 2014 - replace ref 29 (article in a general press newspaper, The NYT) by WHO Ebola Response Team, After Ebola in West Africa — Unpredictable Risks, Preventable Epidemics, N. Engl. J. Med. 375 (2016) 587–596.**

This has now been revised for clarity, referencing the WHO Ebola Response Team NEJM article.

**- Sampling.**

**SLAES database: only confirmed cases in the database resulting in 176 confirmed cases in Kenema ? 143 vs 176: difference due to exclusion of age < 15 years ?**

This has been edited to provide the breakdown of the 164 survivors in the SLAES database. 21 cases were excluded as they were under 15 years of age.

**Controls: sample size calculation is missing; I understand the sampling frame is a 2-stage PPS with first stage selection of 8 EA proportional to the number of survivors in each EA and at the second stage the selection of a fixed number of HH (40) per EA and one individual per HH selected ; questions : what is the total number of EAs in Kenema from 8 EAs were selected ? for the 2d stage, what was the field procedure to select the 40 HH, apart saying it was systematic ? and how was selected the unique participant from the HH, which procedure did you follow ? Clarify the overall sampling procedures.**

We have added information to the sampling section of the paper to clarify these questions.

For sample size, we are generally powered to detect a 15% difference in the prevalence of physical and mental health symptoms with our sample size, with 95% alpha and 80% power, which is less than the actual difference for many (but not all) of the health outcomes.

Regarding the EAs, the total number of EAs in Kenema is approximately 1150 according to the most recent census. However, according to our records of EVD infection, most of these EAs did not have anyone infected with EVD. We limited the eligible EAs to only those that did have at least one person infected, which was 107, from which we selected the 8 EAs for data collection (again, with probability proportionate to the EVD prevalence).

Within each selected EA, we had the overall number of households from the recent census. We obtained maps of each EA and then randomly selected one household within the EA as a starting point, and then selected every nth household afterwards to reach 40 total households.

Finally, the participant within each household was the male or female head of the household, all of whom were over 15 years old.

**Measures: overall, more details are needed to assess the quality of the collected information on the symptoms; is the questionnaire available on-line ? how did you define and diagnose heart problems ? how did you assess duration of symptoms, given the fact it is very difficult in this setting to collect accurate answers on dates ? how did you define severity/symptoms interferring with life ? how did you assess hearing and vision deficiencies ?**

We have further described the survey tool in the “Measures” section, including the wording of each question. An abbreviated version of the survey tool is also uploaded as an attachment. This approach was adapted from previous literature (e.g., **Tozay et al., 2020**). Symptom data was self-reported by EVD survivors and close contacts.

Duration of symptoms in years was estimated by EVD survivors. While this is an approximate, the duration was still significantly higher in EVD survivors compared to community members, with a median of 6.0 (3.0-7.0) years for EVD survivors and 2.0 (1.0-3.0 years) for community members.

In the “Limitations and bias” section, we acknowledge that reports of health symptoms are not a substitution for clinical assessments and measurement with biological markers, and may be subject to, e.g., recall bias and desirability bias. However, we also note that this is not feasible for our population-based study, which has unique benefits in its community-based sample.

Reference: Tozay S, Fischer WA, Wohl DA, Kilpatrick K, Zou F, Reeves E, et al. Long-term Complications of Ebola Virus Disease: Prevalence and Predictors of Major Symptoms and the Role of Inflammation. Clinical Infectious Diseases. 2020;71: 1749–1755. doi:10.1093/cid/ciz1062

**Data analysis: describe data management, data check procedures, missing data handling.**

The team conducted data checks on a daily basis throughout data collection, examining items like potential interviewer effects, the number of interviews per day, and reports of sociodemographic characteristics. Any issues identified in these checks were reported to data collection supervisors to address in the field. We did not have missing values for variables used in this analysis.

**Wealth/data reduction: it seems you retained one component and categorized it in 3 levels after the PCA but the PCA should have led to several components (eigenvalues > 1) ? Did you select the first component?**

The first five components (components with eigenvalues > 1) were extracted and the resulting composite was categorized into 3 levels.

**Analytic methods:**

**"A Poisson regression model was selected due to the right skew of the dependent variable, ...": the dependent variable is a count (nb of symptoms) which is the main reason to use a Poisson model.**

Noted and agreed, this has been revised to clarify that the Poisson regression model (now a negative binomial regression) was used primarily due to the dependent variable consisting of count data.

**"assessed with the Pearson dispersion statistic": did you assess it ? a negative binomial distribution could have been more adequate ?**

Thank you for this comment. To mitigate concerns around the effects of overdispersion and improve the fit, we have now used a negative binomial regression model.

**"Data consisted of independent observations, with no multicollinearity of independent variables, no extreme outliers, a linear relationship between independent variables and the logit of the response variable, and a sufficiently large sample size": any use of goodness-of-fit statistics ?**

Yes. All models were assessed with goodness-of-fit statistics and adjusted to ensure an adequate fit. This has now been detailed in the analysis section, and we are happy to provide additional detail if needed.

**Results**

**- Demo (ref to Table 1):**

**The period (range of dates ) of discharge from ETC of the included survivors should be given.** – The date of discharge is not available for all EVD survivors in our database, but, as mentioned in the manuscript, the survivors included are selected from a full list of remaining survivors, so they should represent the discharge dates among remaining EVD survivors in Kenema District (mid-2014 to 2015).

**Precise in the text the number of participants in both groups.**

This has now been specified in the text above Table 1 and Table 2.

**"Differences between demographics of the EVD survivor and community member participant groups were calculated with independent t-tests and chi square tests, depending on the nature of the dependent variable. T-tests were run to compare the means of age and number of children due to their continuous nature, whereas chi square 213 tests were run for categorical dependent variables.": move to methods.**

This has been taken out of this section and left in the methods section.

**Table 1: give the context in the title(study pop, location, year); some p-values are not in the right column there is a borderline difference in age distribution <60/60+ between survivors and controls (60+ : 4.5% vs 10.0%; Fisher exact p=0.05).**

We have reviewed the paper to ensure that font, spacing, and table formatting is all correct and consistent. The context is now given in the titles of Tables 1 and 2. While a Mann-Whitney test was utilized to determine that the difference between the average ages of the EVD survivor and community member populations was insignificant, the variation in the distribution between age groups is described in the text below Table 2.

**"Differences in ages and genders may reflect limitations of the sampling methodology. Due to the survey data collection in the harvest period at the beginning of the dry season (October-November 2021) [46], middle-aged men may have been working at their farms, while younger and older individuals (primarily female) may have been at home. Gender differentials may also match the demographics of the EVD survivor sample due to similar gender norms, as females are thought to be more likely to be infected with EBOV due to their traditional role as caretakers [35].": there is no difference in gender in table 1 but this paragraph should be moved to the Discussion section.**

This has been removed due to the lack of statistical significance of age and gender measures.

**"Variances in location....Therefore, it cannot be used as a reliable measure of location." : move to discussion.**

We have removed this, as it is irrelevant to the subsequent analysis.

**- Sequelae (ref to Table 2):**

**"They were asked to select...number of children, and wealth status).": move to discussion.**

This has now been removed, as it is also discussed in the methods and discussion sections.

**I suggest to start the paragraph with the grouped symptoms and the Poisson regression (lines 257-260).**

We agree; the additional text before the results of the regression was removed.

**Self-reported...**

We have now edited this to make it clear that symptoms were self-reported and were not assessed by a clinician or associated with biological markers.

**"In addition, binomial logistic regressions...to generate ORs and AORs.": move to methods.**

This has been moved to methods and this additional text has been deleted.

**"spells of terror and panic [AOR = 134.09 (95% CI, 17.41-1032.74), p <0.001] and feeling suddenly scared for no reason [AOR = 64.27 (95% CI, 8.08-511.03), p <0.001]." : very large CI due to only one occurence in the control group, an indication of the goodness of fit is necessary.**

The criteria for goodness of fit (for the logistic regressions, Homer-Lemishow p > 0.05) is now discussed in the analytical methods section. Due to relatively low occurrences of symptoms in the control group for some symptoms, the confidence intervals are large.

**Figure 1 gives the same information and less accurately than Table 2.**

Agreed; Figure 1 has been removed.

**Discussion**

**Very long: more than 6 pages, the discussion on mental health consequences while very important could be shorten.**

We have shortened the discussion section by focusing only on the immediate results from our analysis.

**"Unlike previous research, this study has a comparison group": PREVAIL III included a comparison group.**

We appreciate this comment and have updated the introduction and discussion sections to better describe previous studies (including PREVAIL III), and how this research fits within the broader body of work. In short, we agree with the reviewer that the main contribution of this work is the population-based comparison group.

**although many other studies were restricted to the first few years following the epidemic" : the clinical follow-up in Guinea/Postebogui was 4 years (Ref 36).**

This statement has been revised throughout to reference the other long-term studies after the EVD epidemic, and the Postebogui study was described in lines 348-50.

**"Liberian Ebola Survivor Cohort": not in the References list (see above).**

The Liberian Ebola Survivor Cohort study is reference # 9 (Wohl et al., 2023) and 10 (Tozay et al., 2020).

**Ocular issues : add Ref E.H. Hebert, M.O. Bah, J.F. Etard, M.S. Sow, S. Resnikoff, C. Fardeau, A. Toure, A.N. Ouendeno, I.C. Sagno, L. March, S. Izard, P.L. Lama, M. Barry, E. Delaporte. Ocular Complications in Survivors of the Ebola Outbreak in Guinea, Am. J. Ophthalmol. 175 (2017) 114–121.** [**https://doi.org/10.1016/j.ajo.2016.12.005**](https://doi.org/10.1016/j.ajo.2016.12.005)**.**

Thank you for the suggestion. This has now been referenced.

**Strengths of the study: a population-based comparison is truly a major strength and should be recognized ; it's a clear difference with other studies which used contacts as a comparison group; it's a good reason to detail the sampling methodology.**

We agree with this comment and have updated the introduction, discussion, and sampling sections to describe our approach and contribution.

**"and those who were not surveyed were not notably different in age and gender compared to those who were." : not documented.**

Given the small number of EVD survivors who were not interviewed (n=33), we feel that it is not necessary to formally compare the characteristics of those interviewed with those not interviewed. But we are willing to do so if there is reason to believe otherwise.

**- Limitations.**

**to further discuss selection bias, give the period of discharge, closed to dates of admission in ETC/ETU (see above) ? did recruitment uptake in the survivor cohort change over time during the outbreak ?**

We have a census of all EVD survivors in Kenema District, barring those who have relocated or died prior to survey administration. As a result, the population of survivors eligible for this study represents those of Kenema district, in terms of period of discharge (and other characteristics).

**"... the EVD survivors surveyed were laboratory-verified cases and had certificates": info to be added in the Methods section.**

This has been clarified in the Methods (Sampling and recruitment) section.

**"... responses were all self-reported and not verified by a physician": to be discussed in relation to recall bias and information bias.**

We have added a note about this bias to the limitations section.

**"... with a high statistical power due to its robust, population based sample and comparison group": power is not assessed, so the point is purely declarative.**

We agree that, since other studies had larger sample sizes than ours, this is not an advantage of our study. We have deleted this statement as a result.

**a comment on the absence of any objective biological markers should be added.**

This has been integrated into the point about self-reporting of perceived symptoms and associated biases in lack of confirmation by a physician or objective biological markers.

**Conclusion**

**yes. I agree on a survivors care package available through the current health system after the end of the international aid. It falls within the global strengthening of the health system of the low-income countries.**

Agreed
