# Supplementary material for: Long-term physical and mental health outcomes of Ebola Virus Disease survivors in Kenema District, Sierra Leone: A cross-sectional survey
Source: PLOS Glob Public Health. 2024 Nov 4;4(11):e0003421. doi: 10.1371/journal.pgph.0003421 (PMC11534246; doi:10.1371/journal.pgph.0003421)
Supplement: Attachment — Submitted filename: Review.docx [file pgph.0003421.s006.docx]

Long-term physical and mental health outcomes of Ebola Virus Disease survivors in Kenema District, Sierra Leone: a cross-sectional survey.

Overall Comment

This is an interesting paper on the long term health outcomes of Ebola survivors in Sierra Leone. The authors convey the case study in the language of EVD evolution, and multilevel factorial dynamics characterised by iteration and emergence. While the paper is an interesting contribution to the literature, the revised version has two main weaknesses. Firstly, in terms of methods, and nature of ‘data collection’ and secondly in terms of empirical/description detail.

Firstly, the methodology is unclear and in some sub-sections contradictory. The data collection reads as qualitative, I doubt the use of the term ‘interviews’ as the appropriate data collection approach used in this study, a study that is quantitatively analyzed and presented. Forms of interviews, discussions, and observations are qualitative data collection methods/approaches and therefore should be analysed as such, thematically, rounded-theory etc…

On the other hand, if the data collection of this study involved reading out surveys to participants and reporting their responses in the survey sheet, that is not an interview, it is a standard survey approach for participants who cannot read. So, I would urge that, if the data collection strategy involved distribution and completion of a survey, as it appears to be, then do not refer to that process as interviews are done.

However, if actual interviews were conducted, then you would need to provide a description of the type of interviews done, suitability for this study, theme/interview guides, variations of participants, heterogeneity, positionality/ and qualitative analysis plan. This would also nullify the entire result presentation in this study. The analysis, whether grounded or thematic should be explained.

In a mixed-methods approach, which may involve both interviews (qualitative) and Survey quantitative) the interpretation of both data and synergies in both sets of findings be presented and discussed.

Secondly, on the empirical/description basis of the physical symptoms: A clear distinction of symptoms/signs and sequelae of EVD should be made. Were these symptoms/signs noted/reported during EVD and therefore have progressed to chronicity (now Sequelae) or were these symptoms/signs intermittent occurrences? I see these terms used interchangeably throughout the manuscript but would be good to differentiate. Symptoms like fever and diarrhoea can be mostly considered acute and or intermittent precipitated by an ongoing process. It would be a big claim to consider the persistence of such symptoms for 7 years after the initial EVD infection.

The paper presents limited evidence of how the study’s findings are different from similarly studies done in 5 years period after EVD occurrence. Would an additional year (6 or 7) compared to that of 5-year studies, identify new symptoms or simply track progression/improvement of the same symptoms studied throughout the previous 5 years? And how does the local health system structure to address expected age-related conditions such as joint pain etc, access to symptomatic treatment and specialist services for chronic sequalae in the discussion section.

I hope these comments are useful and encourage the authors to further strengthen this interesting piece.

Specific Comments

1. Page 4, Line 79: The statement ‘…with joint and muscle pain, fever, headaches….’ should not report fever as sequelae.
2. Page 7, Line 160: clarify whether “interview” refers to qualitative interviews, as data collection. Please change and check throughout for consistency.
3. Page 7, Line 160-164: is this the Kish grid approach, and household selection strategy? How feasible is this in Sierra Leone and Kenema in identifying the specific household?
4. Page 8, Line 183: "Measures". A table of symptoms and sequelae, definitions and criteria for inclusion as EVD cause would improve this sub-section.
5. Page 9, Line 194: Did you use both statistical software for your analysis or just one (which one)?
6. Page 9, Line 205: The phrase “ownership of electricity” is not clear. Please rephrase.
7. Page 9, Line 213: How was missing data and multicollinearity handled.
8. Page 20, Line 399: Change ‘six -years’ to ‘more than Six-year’
9. Page 21, Line 432: The statement ‘This is an interesting finding’, could rephrased more specifically. Is ‘this’ referring to all sequelae previously listed or one in particular?
10. Reference 3 may be incomplete, please review
11. Please review references 37 and 57
12. Figure 'Duration of Symptoms' could be presented differently. Grouping all symptoms (one or more) and plotting that to number of participants could be misleading. I wound not expect fever to be present for 6+ years but Joint pain might be which could be non-EVD related. Different acute/intermittent symptoms and Chronic sequelae, to be plot with participants.
