# Supplementary material for: Long-term physical and mental health outcomes of Ebola Virus Disease survivors in Kenema District, Sierra Leone: A cross-sectional survey
Source: PLOS Glob Public Health. 2024 Nov 4;4(11):e0003421. doi: 10.1371/journal.pgph.0003421 (PMC11534246; doi:10.1371/journal.pgph.0003421)
Supplement: Attachment — Submitted filename: Response to Reviewers 9.20.2024.docx [file pgph.0003421.s007.docx]

**Ref.: PGPH-D-24-00262**

**Long-term physical and mental health outcomes of Ebola Virus Disease survivors in Kenema District, Sierra Leone: a cross-sectional survey**

We thank the editor and reviewers for their thoughtful comments and feedback. We have listed the editor’s and both reviewers’ comments in bold with our responses in plain font underneath each individual comment.

**General Comments**

1. **In the ethics statement in the Methods, you have specified that verbal consent was obtained. Please provide additional details regarding how this consent was documented and witnessed, and state whether this was approved by the IRB**

This has been described on pg. 10, lines 223-229.

2. **Please include a complete copy of PLOS’ questionnaire on inclusivity in global research in your revised manuscript. Our policy for research in this area aims to improve transparency in the reporting of research performed outside of researchers’ own country or community. The policy applies to researchers who have travelled to a different country to conduct research, research with Indigenous populations or their lands, and research on cultural artefacts. The questionnaire can also be requested at the journal’s discretion for any other submissions, even if these conditions are not met.  Please find more information on the policy and a link to download a blank copy of the questionnaire here:** [**https://journals.plos.org/globalpublichealth/s/best-practices-in-research-reporting**](https://journals.plos.org/globalpublichealth/s/best-practices-in-research-reporting)**. Please upload a completed version of your questionnaire as Supporting Information when you resubmit your manuscript.**

This has been submitted as Supporting Information.

3. **Please review your reference list to ensure that it is complete and correct. If you have cited papers that have been retracted, please include the rationale for doing so in the manuscript text, or remove these references and replace them with relevant current references. Any changes to the reference list should be mentioned in the rebuttal letter that accompanies your revised manuscript. If you need to cite a retracted article, indicate the article’s retracted status in the References list and also include a citation and full reference for the retraction notice.**
   The reference list has been reviewed and it is complete and correct. None of the papers used have been retracted.
4. **Firstly, the methodology is unclear and in some sub-sections contradictory. The data collection reads as qualitative, I doubt the use of the term ‘interviews’ as the appropriate data collection approach used in this study, a study that is quantitatively analyzed and presented. Forms of interviews, discussions, and observations are qualitative data collection methods/approaches and therefore should be analysed as such, thematically, rounded-theory etc…

   On the other hand, if the data collection of this study involved reading out surveys to participants and reporting their responses in the survey sheet, that is not an interview, it is a standard survey approach for participants who cannot read. So, I would urge that, if the data collection strategy involved distribution and completion of a survey, as it appears to be, then do not refer to that process as interviews are done.**

**However, if actual interviews were conducted, then you would need to provide a description of the type of interviews done, suitability for this study, theme/interview guides, variations of participants, heterogeneity, positionality/ and qualitative analysis plan. This would also nullify the entire result presentation in this study. The analysis, whether grounded or thematic should be explained.**

**In a mixed-methods approach, which may involve both interviews (qualitative) and Survey quantitative) the interpretation of both data and synergies in both sets of findings be presented and discussed.**

To be clear, our approach was a quantitative survey and there were no qualitative aspects to this research. We have clarified this by stating that our work was quantitative in the introduction, methods, and discussion section of this manuscript.

The term “interview” equally applies to both quantitative and qualitative data collection approaches. Below we list several textbooks that refer to quantitative data collection as “interviews,” so this term does not apply only to qualitative research.

Ultimately, we want to find a balance between being clear about our data collection and analysis approach, but also using the standard terminology in the field. To do so, we have stated that our data collection and analysis was quantitative.

References

Fowler Jr, F. J. (2013). *Survey research methods*. Sage publications.

Rea, L. M., & Parker, R. A. (2014). *Designing and conducting survey research: A comprehensive guide*. John Wiley & Sons.

Wrench, J. S., Thomas-Maddox, C., Richmond, V. P., & McCroskey, J. C. (2008). *Quantitative research methods for communication: A hands-on approach.* Oxford University Press, Inc..

Zhou, X., & Jiu, B. (2008). *A practical guide to quantitative finance interviews*. Createspace Independent Publishing Platform.

5. **Secondly, on the empirical/description basis of the physical symptoms: A clear distinction of symptoms/signs and sequelae of EVD should be made. Were these symptoms/signs noted/reported during EVD and therefore have progressed to chronicity (now Sequelae) or were these symptoms/signs intermittent occurrences? I see these terms used interchangeably throughout the manuscript but would be good to differentiate. Symptoms like fever and diarrhoea can be mostly considered acute and or intermittent precipitated by an ongoing process. It would be a big claim to consider the persistence of such symptoms for 7 years after the initial EVD infection.**

We appreciate this point. “Sequelae” has been changed to “signs and symptoms” throughout, as this was a cross-sectional survey and cannot establish chronicity.

6. **The paper presents limited evidence of how the study’s findings are different from similarly studies done in 5 years period after EVD occurrence. Would an additional year (6 or 7) compared to that of 5-year studies, identify new symptoms or simply track progression/improvement of the same symptoms studied throughout the previous 5 years? And how does the local health system structure to address expected age-related conditions such as joint pain etc, access to symptomatic treatment and specialist services for chronic sequalae in the discussion section.**

We compare our studies to those conducted two to six years after the epidemic on page 6, lines 364-375. Compared to other studies conducted four to six years after the epidemic, this study found similar symptoms but at higher rates (e.g., 75.5% of EVD survivors had joint pain in this study, compared to 39.3% of EVD survivors six years after discharge in the Liberian Ebola Survivor Cohort).

Many chronic symptoms, such as joint pain, are not effectively treated in many parts of Africa due to limited resources and conflicting priorities. This has been described on page 19, lines 436-441.

**Specific Comments**

1. **Page 4, Line 79: The statement ‘…with joint and muscle pain, fever, headaches….’ should not report fever as sequelae.**

Noted, this has now been revised.

2. **Page 7, Line 160: clarify whether “interview” refers to qualitative interviews, as data collection. Please change and check throughout for consistency**.

We have clarified throughout the manuscript that our analysis only includes quantitative data.

3. **Page 7, Line 160-164: is this the Kish grid approach, and household selection strategy? How feasible is this in Sierra Leone and Kenema in identifying the specific household?**

A Kish grid is used to randomly select an eligible household member. But for our survey, we selected the head of household, because the Ebola survivors were disproportionately heads of household themselves. So if we had randomly selected a household member, this would not have matched the Ebola survivors sample. However, the selection of households within enumeration areas (EAs) was done randomly. To do so, we used the number of households in the EA from the 2020 census and obtained maps of the selected EAs. We then selected one household randomly as a starting point, and then selected every *nt*h household after this starting point to obtain a final number of 40 households per EA. This is described on page 6 of the manuscript.

4. **Page 8, Line 183: "Measures". A table of symptoms and sequelae, definitions and criteria for inclusion as EVD cause would improve this sub-section.**

We appreciate the need to be clear about our selection of EVD symptoms used in this research. However, our studies follows several others that have previously justified the choice of symptoms. We have added a statement in the “measures” section stating that “our selection of symptoms measured in this study is based on similar previous studies (Tozay et al., 2020; Wohl et al., 2023; Etard et al., 2017; The Prevail III Study Group, 2019).

References

Tozay S, Fischer WA, Wohl DA, Kilpatrick K, Zou F, Reeves E, et al. Long-term Complications of Ebola Virus Disease: Prevalence and Predictors of Major Symptoms and the Role of Inflammation. Clinical Infectious Diseases. 2020;71: 1749–1755. doi:10.1093/cid/ciz1062

Wohl DA, Fischer WA, Mei W, Zou F, Tozay S, Reeves E, et al. Post-Ebola Symptoms 7 Years After Infection: The Natural History of Long Ebola. Clinical Infectious Diseases. 2023;76: e835–e840. doi:10.1093/cid/ciac732

Etard J-F, Sow MS, Leroy S, Touré A, Taverne B, Keita AK, et al. Multidisciplinary assessment of post-Ebola sequelae in Guinea (Postebogui): an observational cohort study. The Lancet Infectious Diseases. 2017;17: 545–552. doi:10.1016/S1473-3099(16)30516-3

The PREVAIL III Study Group. A Longitudinal Study of Ebola Sequelae in Liberia. N Engl J Med. 2019;380: 924–934. doi:10.1056/NEJMoa1805435

5. **Page 9, Line 194: Did you use both statistical software for your analysis or just one (which one)?**

Initially we started by using SPSS for this analysis. But we later changed to Stata, and re-did all of our analysis in Stata (and conducted all later analysis in Stata). So in the manuscript we have just stated that we used Stata, since there was no analysis that we did only in SPSS.

6. **Page 9, Line 205: The phrase “ownership of electricity” is not clear. Please rephrase.**

This has now been revised to “presence of electricity or a radio in their home.”

7. **Page 9, Line 213: How was missing data and multicollinearity handled.**

There was no multicollinearity (pg. 10, lines 216-217) or missing data (pg. 7, lines 154-155).

8. **Page 20, Line 399: Change ‘six -years’ to ‘more than Six-year’**

Noted; this has now been changed throughout the article.

9. **Page 21, Line 432: The statement ‘This is an interesting finding’, could rephrased more specifically. Is ‘this’ referring to all sequelae previously listed or one in particular?**

This has been removed, and findings in this study compared to previous studies are described on pg.17, lines 352-358.

10. **Reference 3 may be incomplete, please review**

This has been reviewed and it is complete.

11. **Please review references 37 and 57**

This has been reviewed and updated.

12. **Figure 'Duration of Symptoms' could be presented differently. Grouping all symptoms (one or more) and plotting that to number of participants could be misleading. I wound not expect fever to be present for 6+ years but Joint pain might be which could be non-EVD related. Different acute/intermittent symptoms and Chronic sequelae, to be plot with participants.**

We appreciate this comment. Although the symptoms included in this figure are different in nature, we feel that it’s important to show that EVD survivors experience many symptoms for a long period of time, longer than non EVD survivors. We intend to expand this research to look more closely at specific symptoms in the future, but this is beyond the scope of the current research.
